# Supplementary material for: Rhodium-Catalyzed Trans-Bis-Silylation Reactions of 2-Ethynyl-3-pentamethyldisilanylpyridines
Source: Molecules. 2023 Apr 7;28(8):3284. doi: 10.3390/molecules28083284 (PMC10142085; doi:10.3390/molecules28083284)
Supplement: Supplementary file 1 [file molecules-28-03284-s001.zip › molecules-2238405-supplementary.pdf]

Supplementary Materials

Rhodium-catalyzed *trans*-bis-silylation reactions of 2-ethynyl-3-pentamethyldisilanylpyridines

Akinobu Naka,*[1] and Hisayoshi Kobayashi*[2]

[1]Department of Life Science, Kurashiki University of Science and the Arts, Nishinoura, Tsurajima-cho, Kurashiki, Okayama 712-8505, Japan

[2] Professor Emeritus Kyoto Institute of Technology, Matsugasaki, Kyoto 606-8585, Japan

Contents

1. NMR Spectra……………………………………………………………………….S2-S7
2. Optimized structures for all LM's and TS's for *trans*-bis-silylation product…………S8-S22
3. Scheme S1………………………………………………………………………….S23
4. Optimized structures for all LM's and TS's for *cis*-bis-silylation product………..…. S24-S31

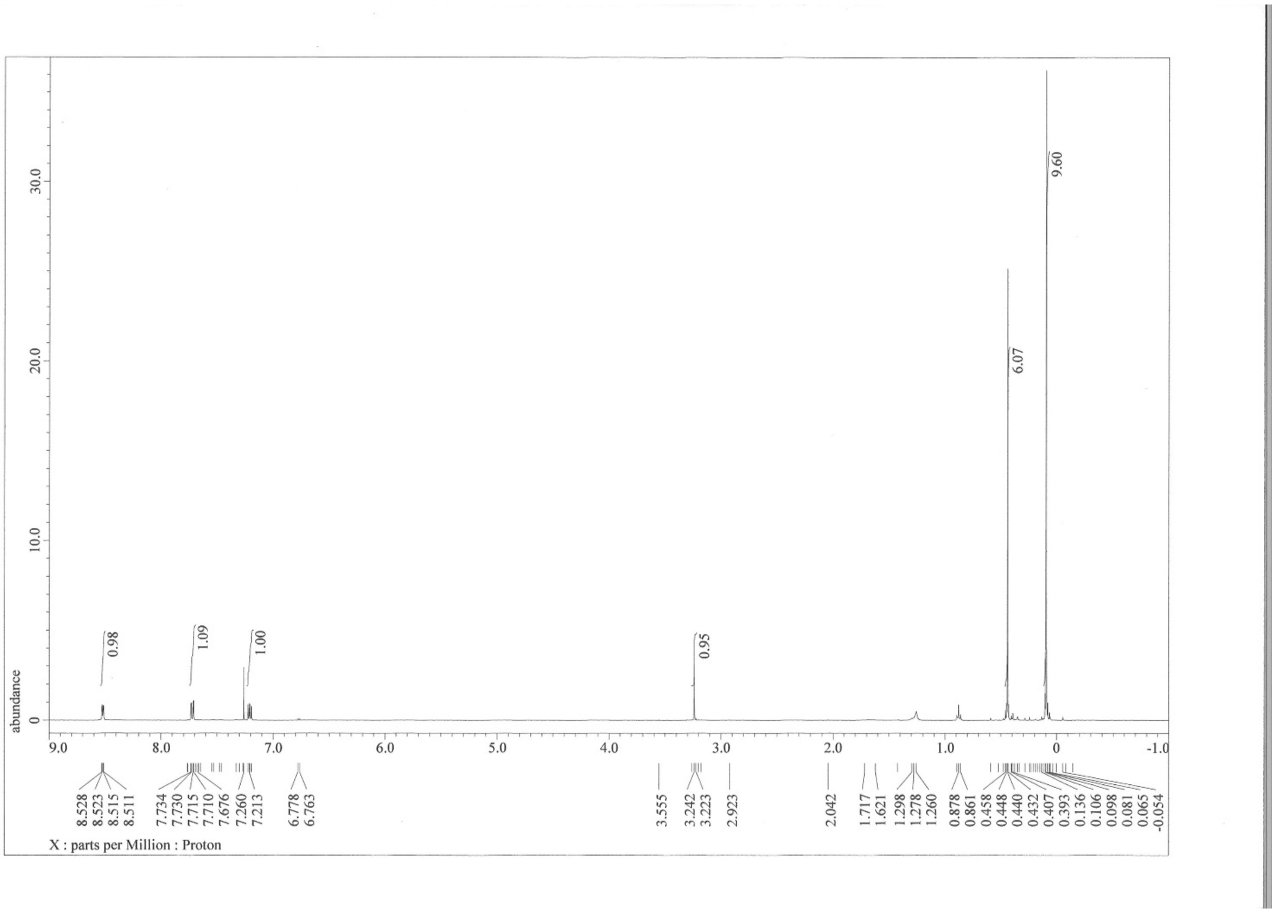

Figure S1. ^{1}H NMR spectrum of **2** in $CDCl_3$.

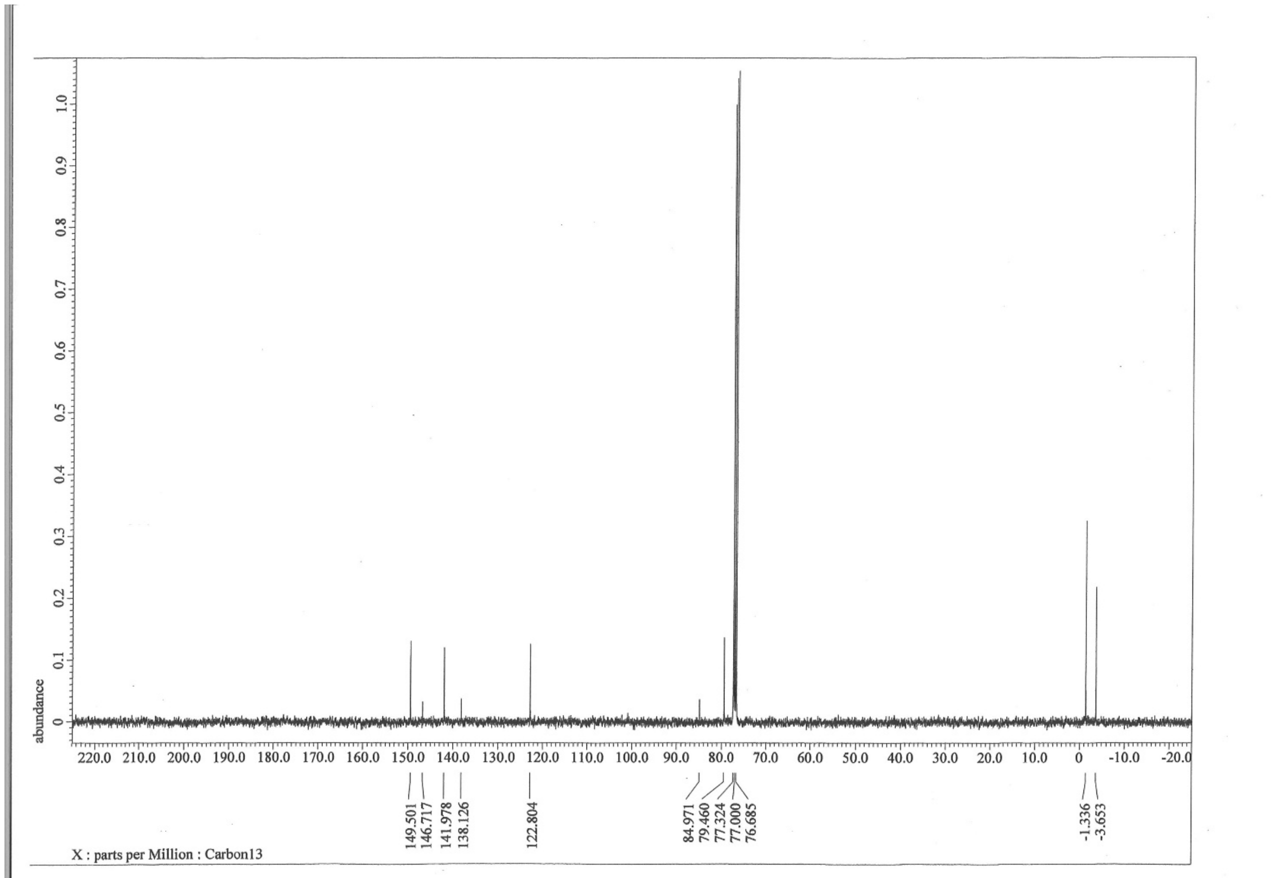

Figure S2. ^{13}C NMR spectrum of **2** in $CDCl_3$.

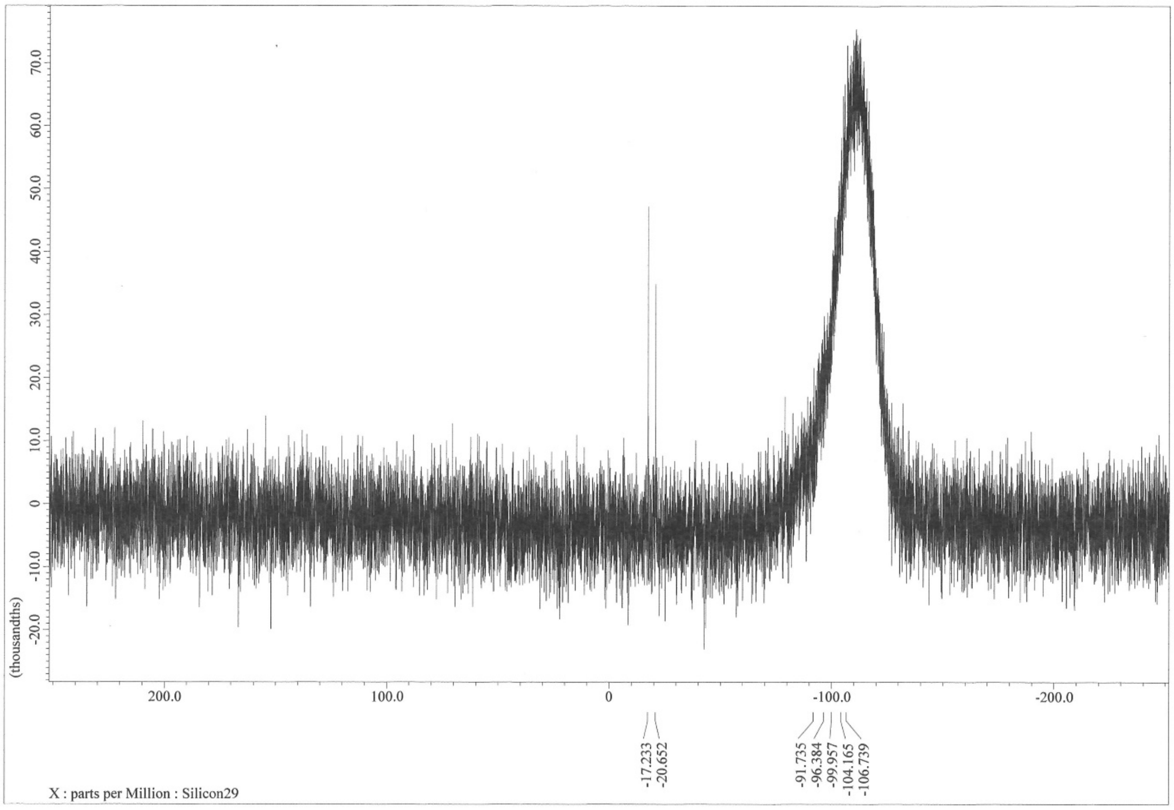

Figure S3. ^{29}Si NMR spectrum of **2** in $CDCl_3$.

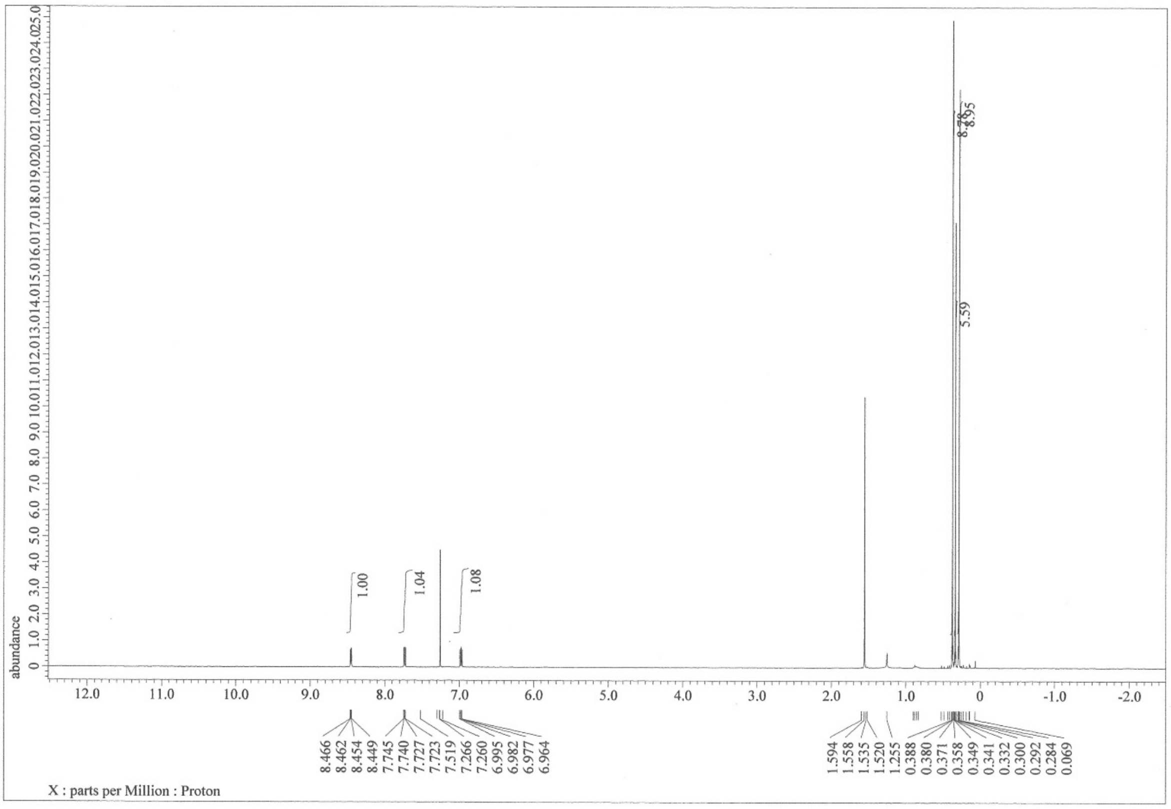

Figure S4. ^{1}H NMR spectrum of **3** in $CDCl_3$.

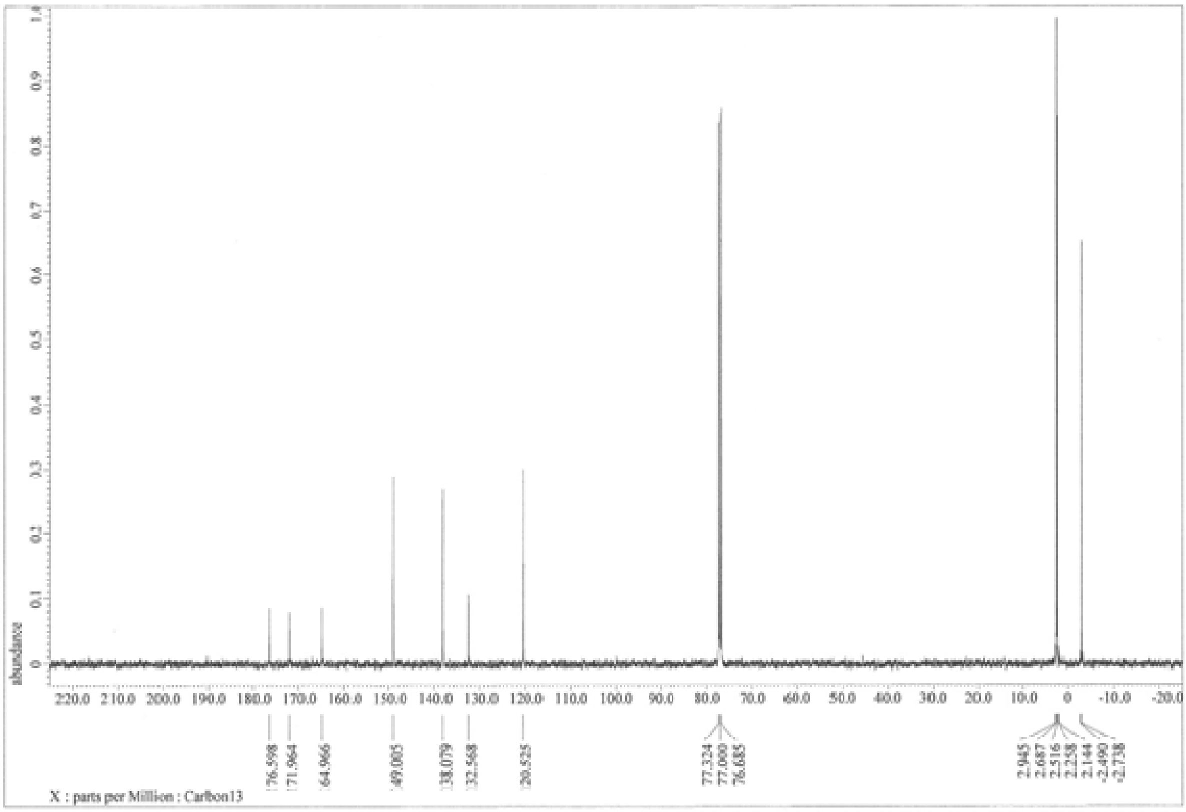

Figure S5. ^{13}C NMR spectrum of **3** in $CDCl_3$.

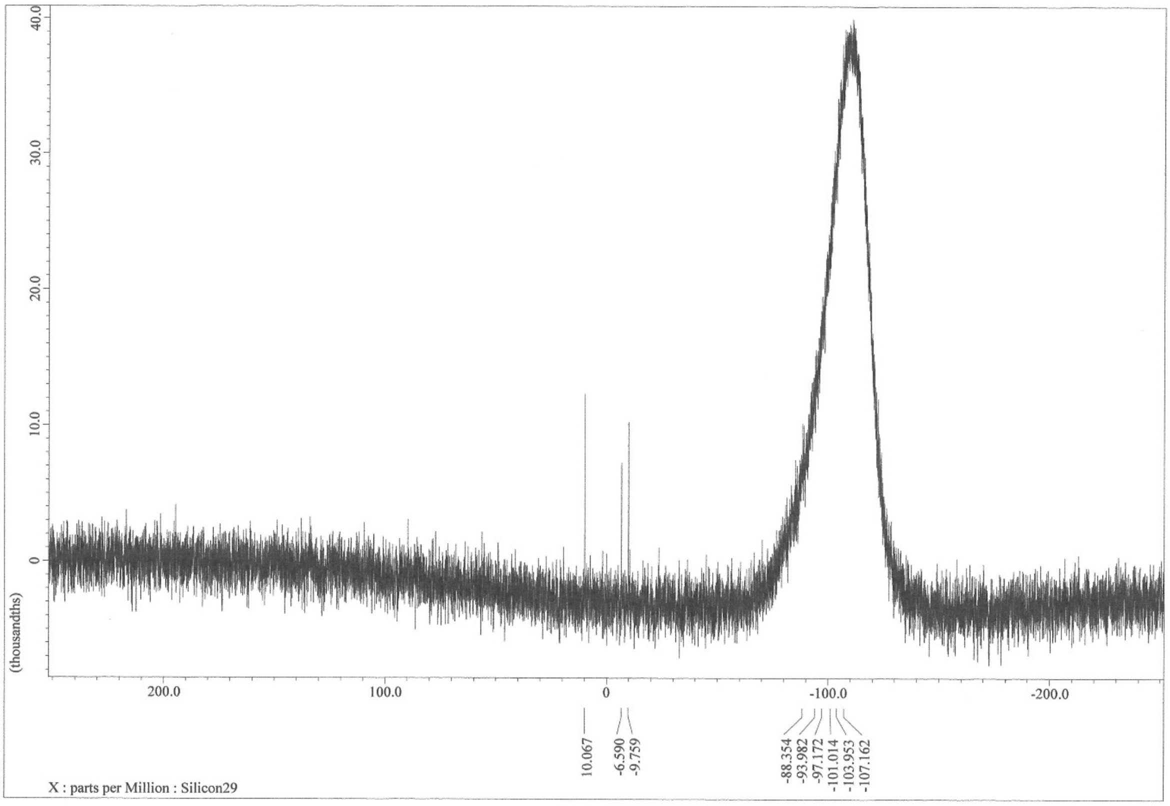

Figure S6. ^{29}Si NMR spectrum of **3** in $CDCl_3$.

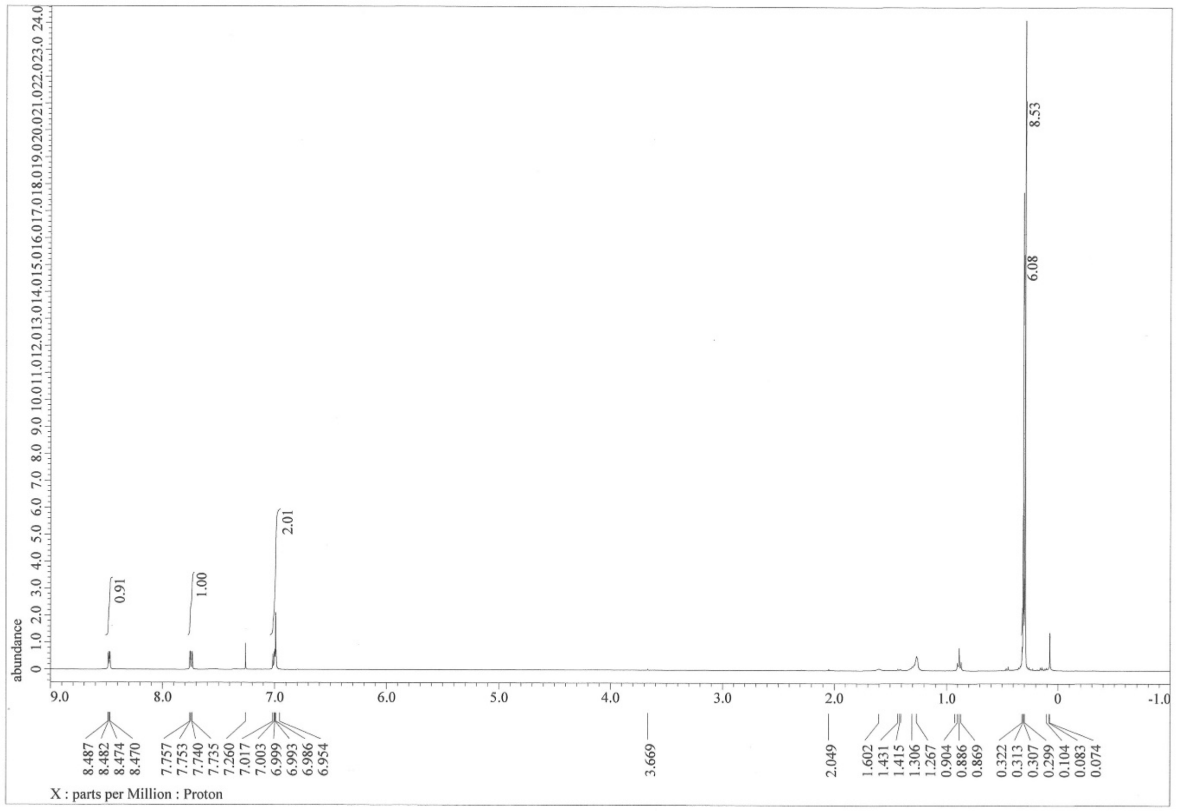

Figure S7. ^{1}H NMR spectrum of **4** in $CDCl_3$.

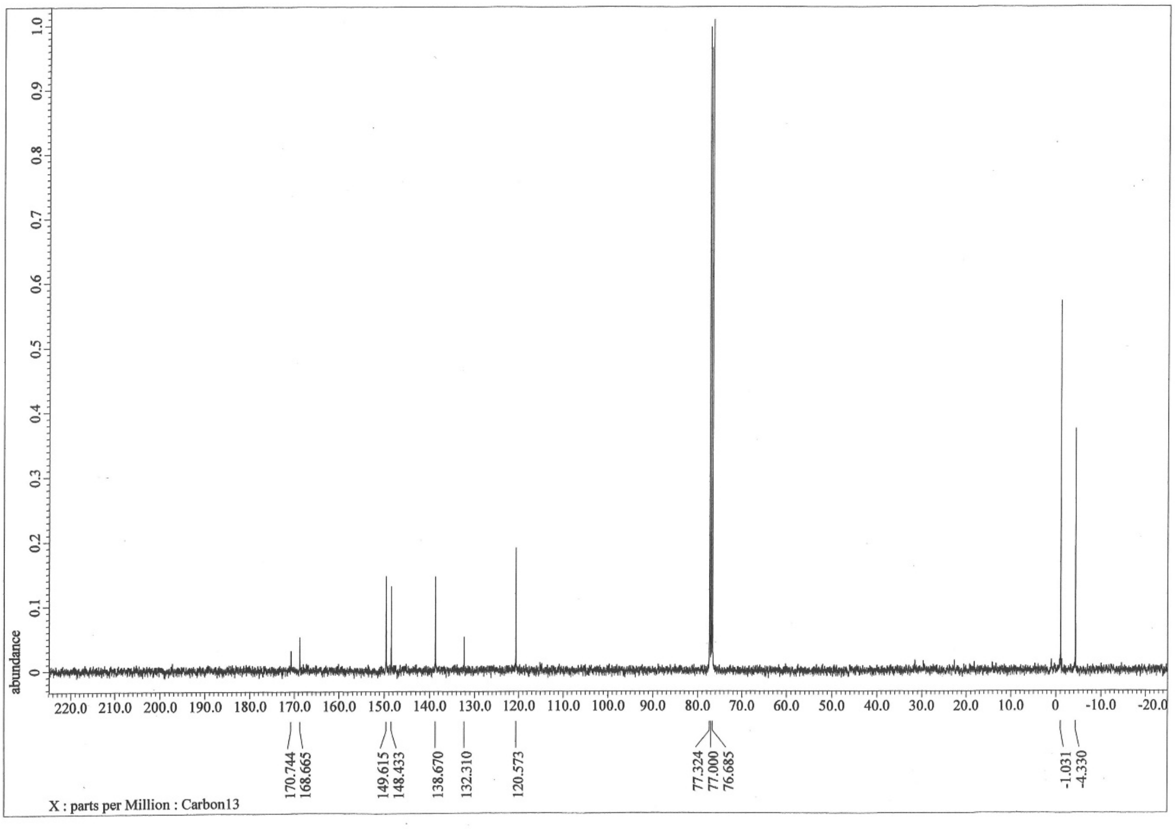

Figure S8. ^{13}C NMR spectrum of **4** in $CDCl_3$.

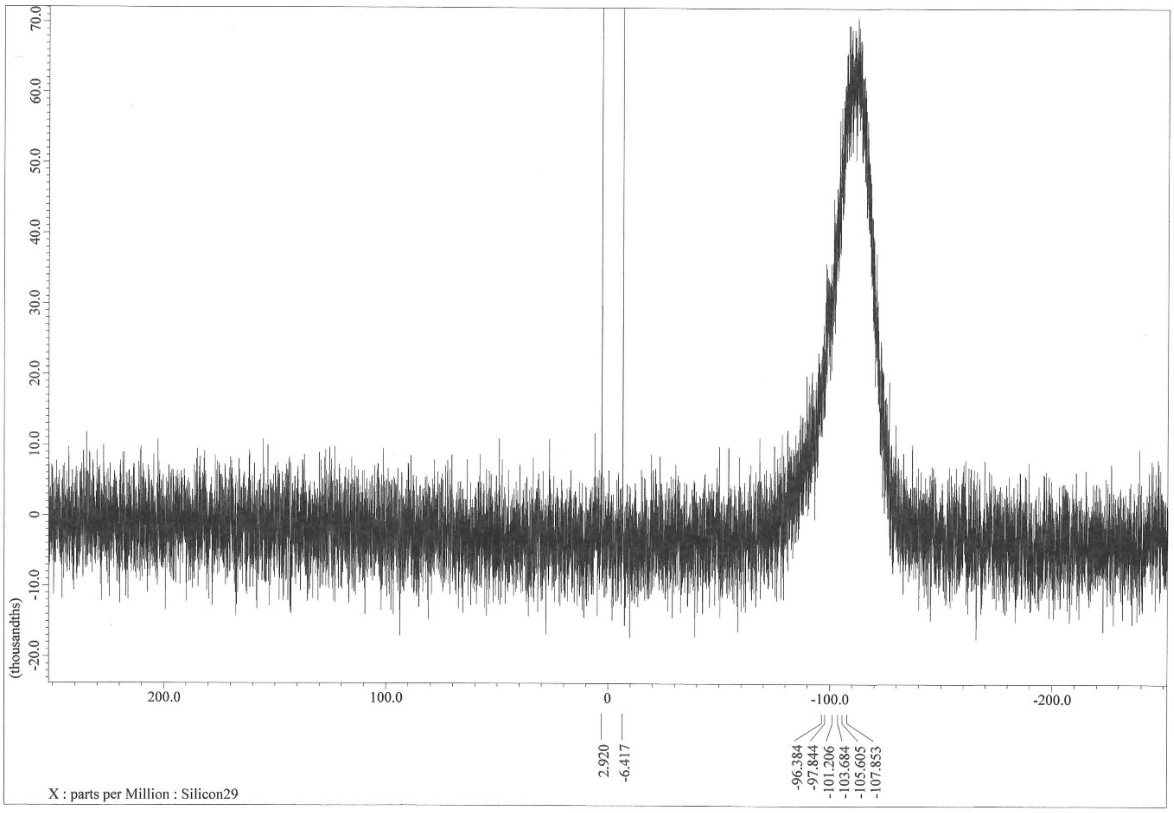

Figure S9. ^{29}Si NMR spectrum of **4** in $CDCl_3$.

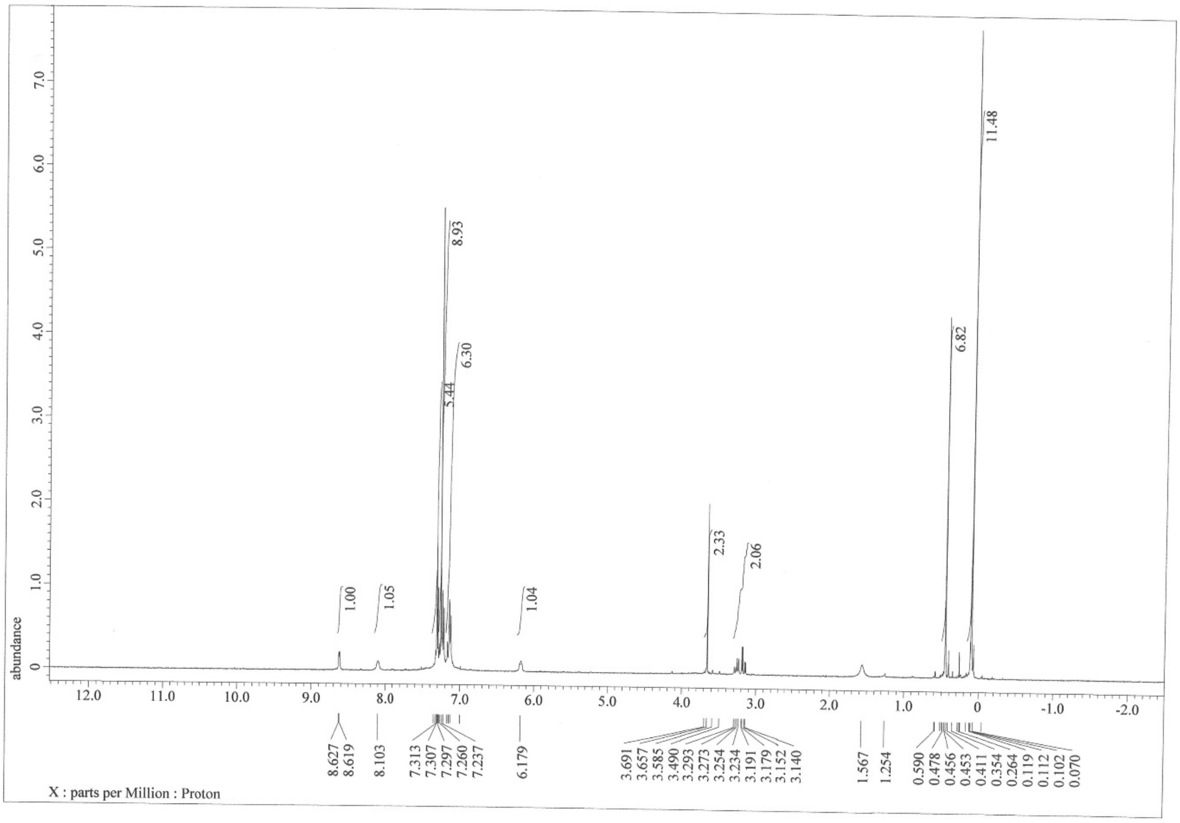

Figure S10. ^{1}H NMR spectrum of **6** in $CDCl_3$.

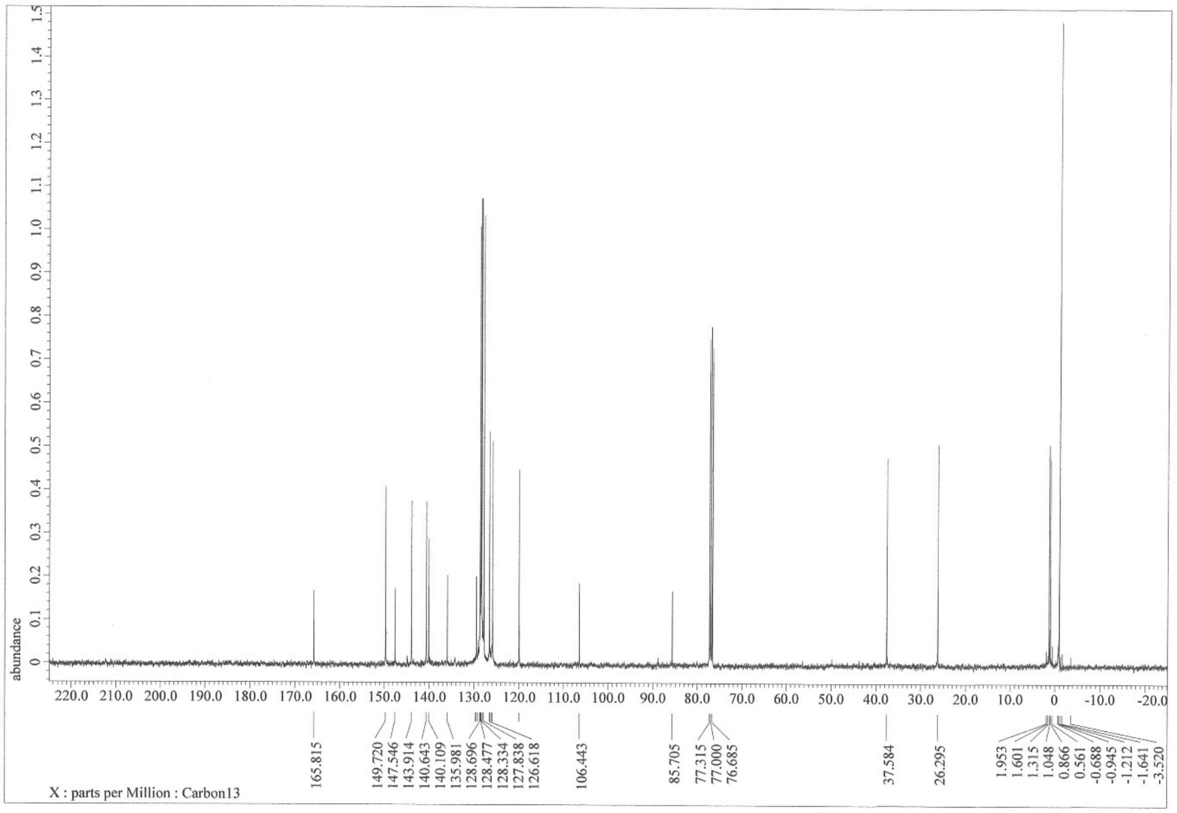

Figure S11. ^{13}C NMR spectrum of **6** in $CDCl_3$.

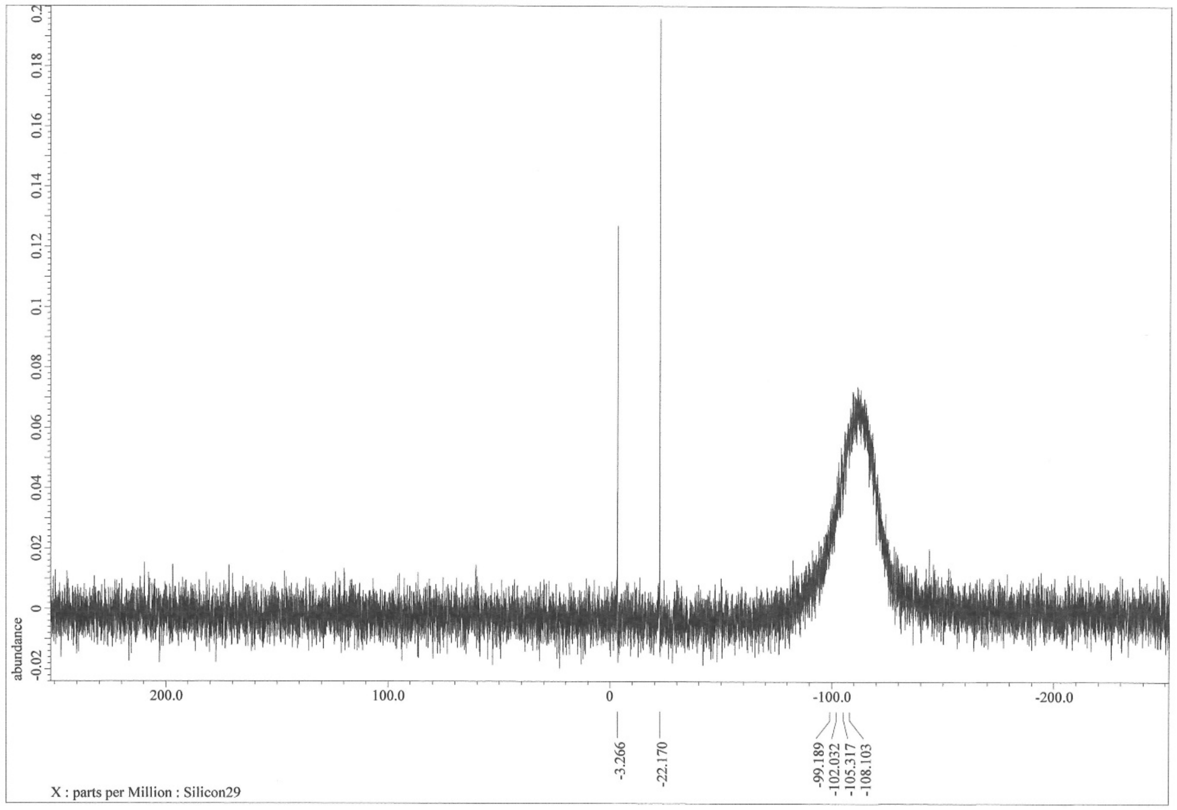

Figure S12. ^{29}Si NMR spectrum of **6** in $CDCl_3$.

Optimized structures for all LM's and TS's on the trans route with RhCl(CO) model.

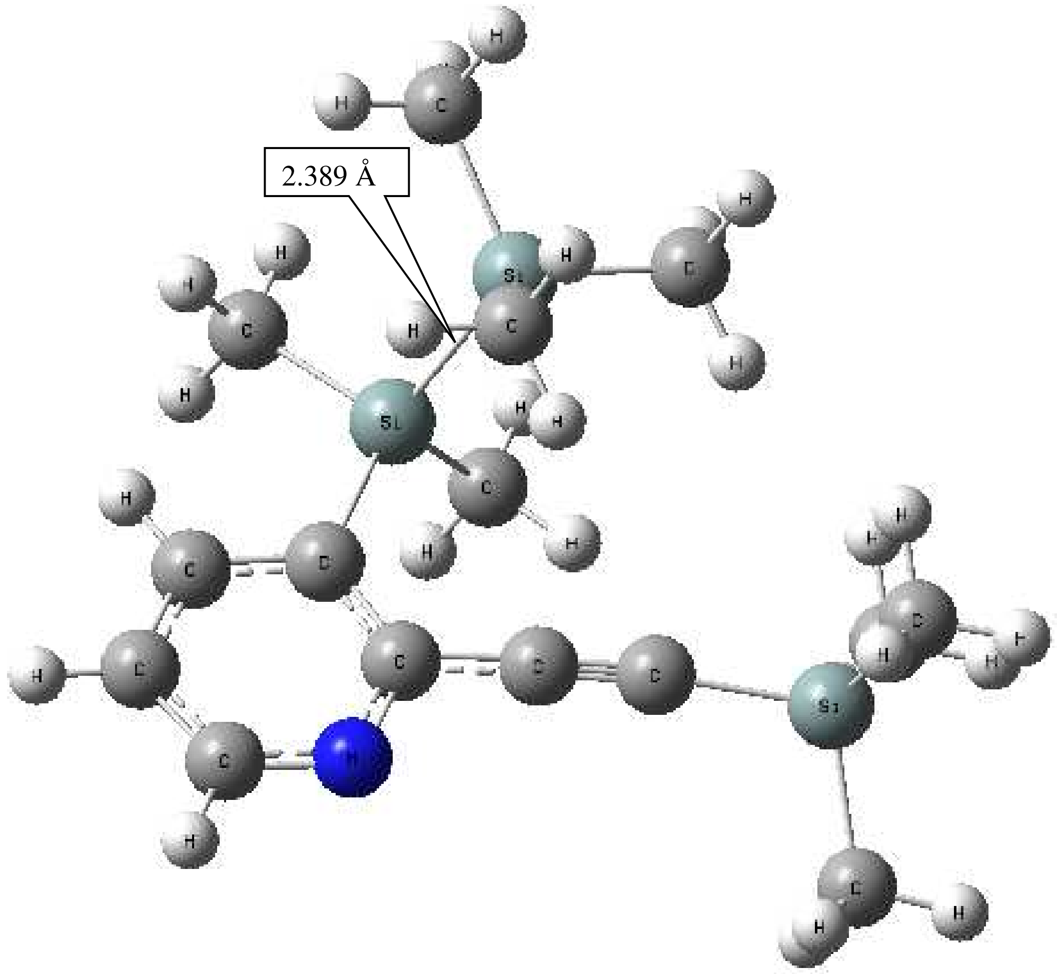

Figure S13. Optimized structure of **1**.

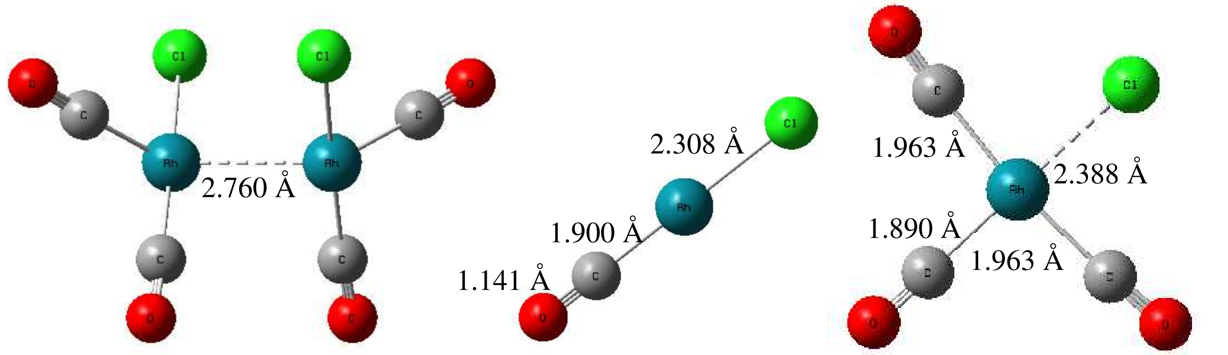

Figure S14. Optimized structures for dimer, and decomposed and disproportionated monomer Rh complexes. $(RhCl(CO)_2)_2$ (left), RhCl(CO) (center) and $RhCl(CO)_3$ (right). The sum of energies of isolated **1** and $(RhCl(CO)_2)_2$ was adopted as the reference energy throughout the reactions.

From **Figures S15** to **S21**, LM and TS structures on trans route with RhCl(CO) model are drawn.

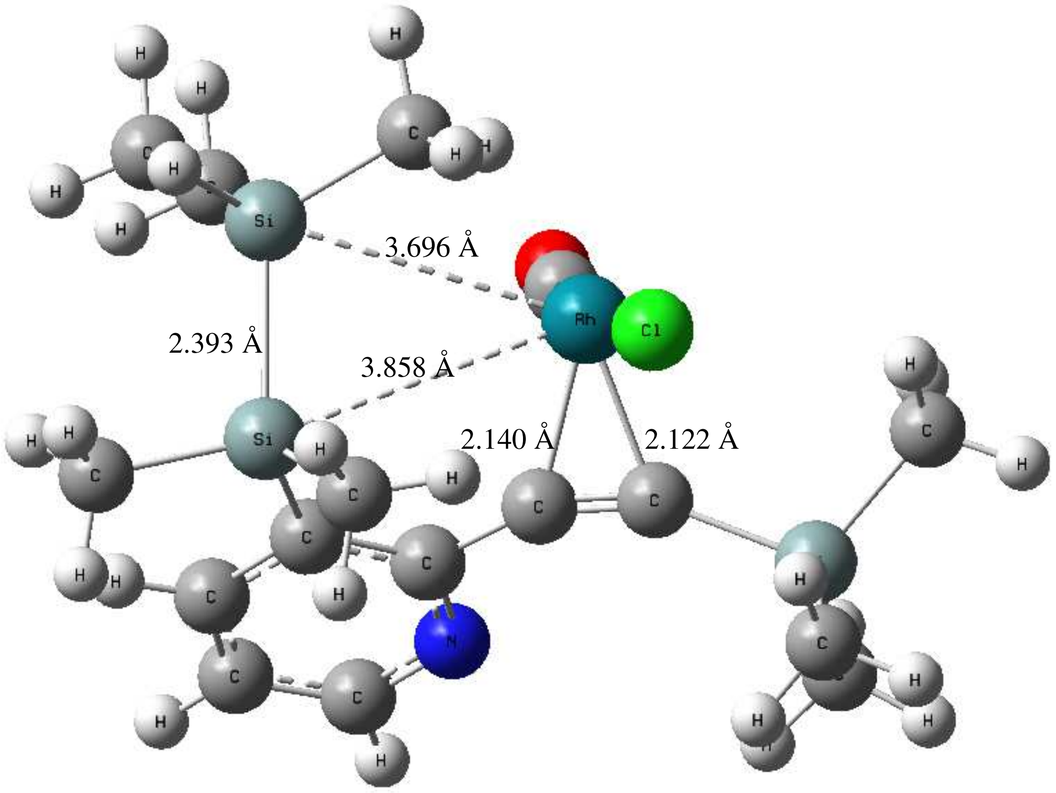

Figure S15. Optimized structure of **model 0(1)**. **Model 0** is a combined structure of **1** and RhCl(CO).

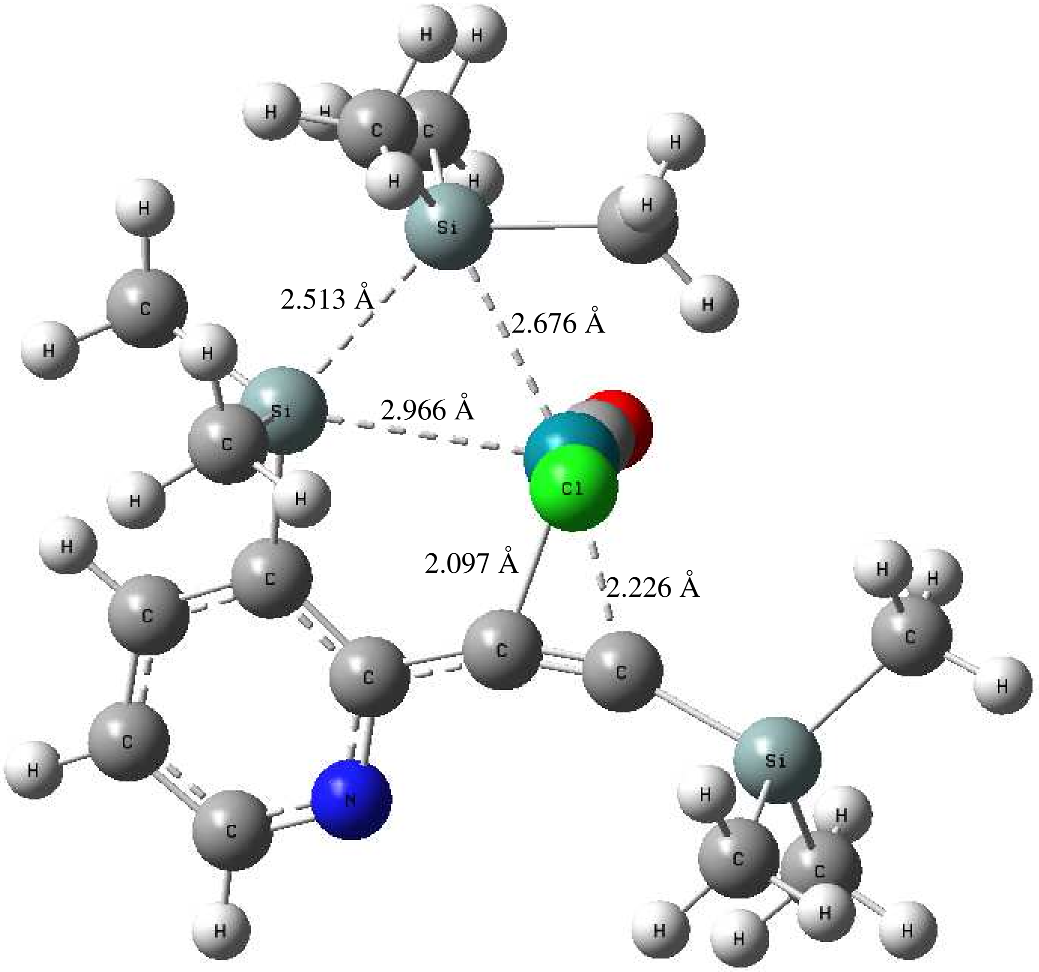

Figure S16. Optimized structure of **TS 0-1(1)**. **TS 0-1(1)** is the TS between **model 0(1)** and **model 1(1)** with RhCl(CO) model.

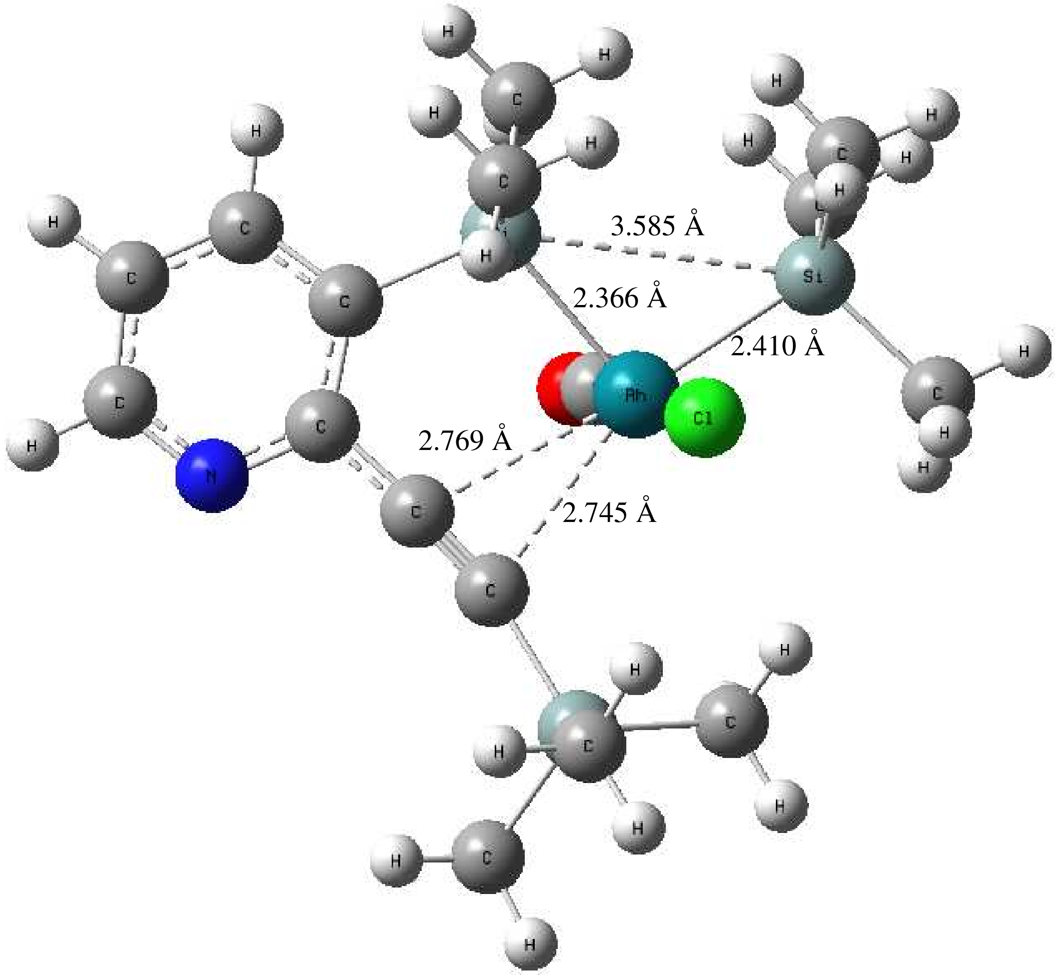

Figure S17. Optimized structure of **model 1(1)** with RhCl(CO) model.

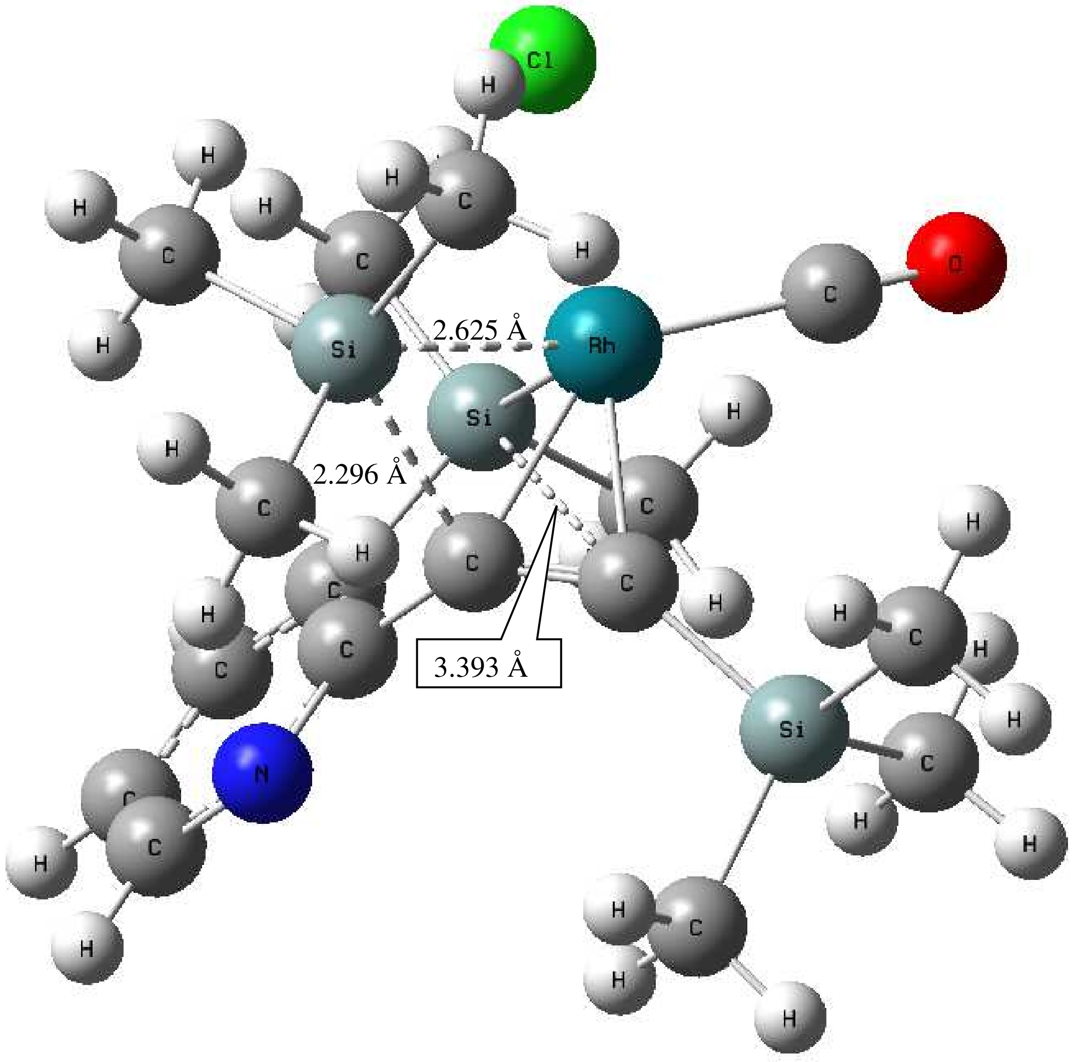

Figure S18. Optimized structure of **TS 1-2(1)** with RhCl(CO) model. **TS 1-2(1)** is the TS between **model 1(1)** and **model 2(1)**.

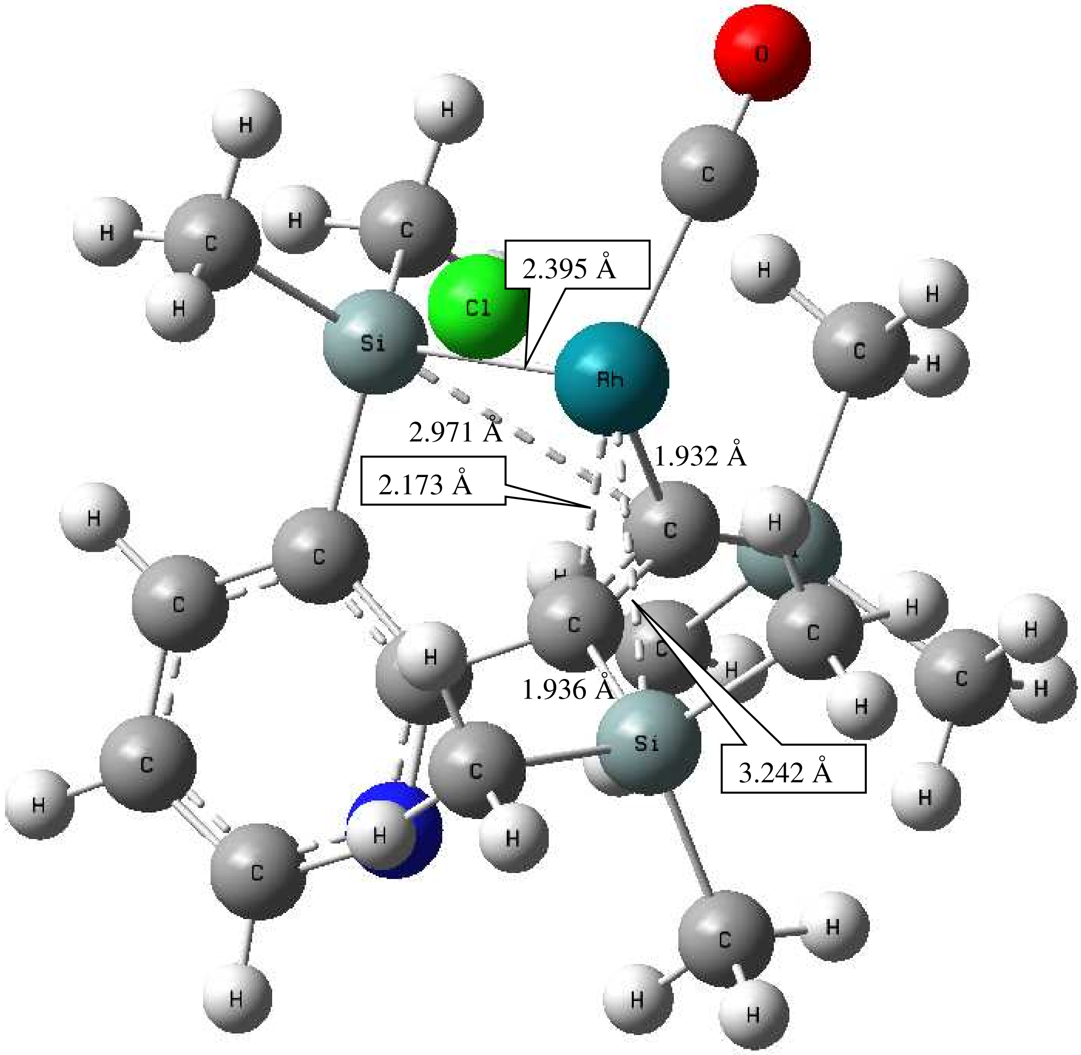

Figure S19. Optimized structure of **model 2(1)** with RhCl(CO) model.

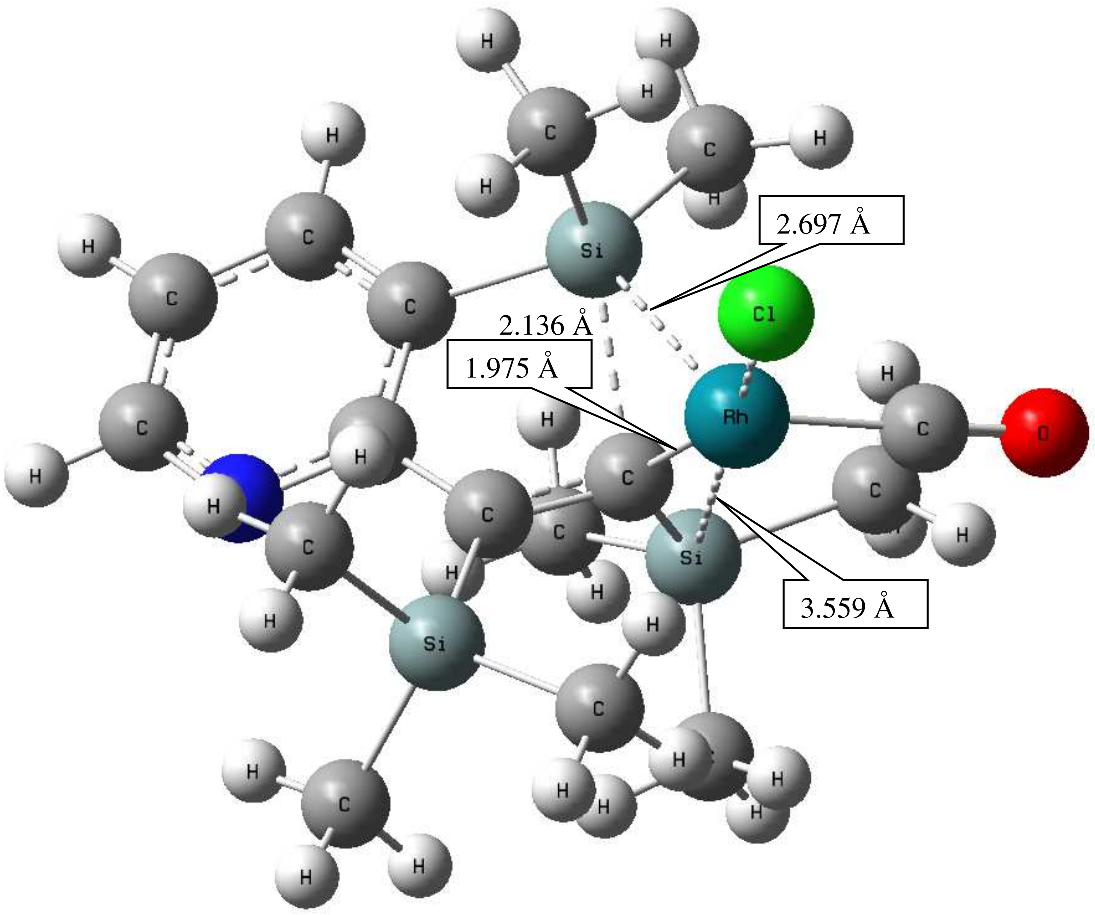

Figure S20. Optimized structure of **TS 2-3(1)** with RhCl(CO) model. **TS 2-3(1)** is the TS between **model 2(1)** and **model 3(1)**.

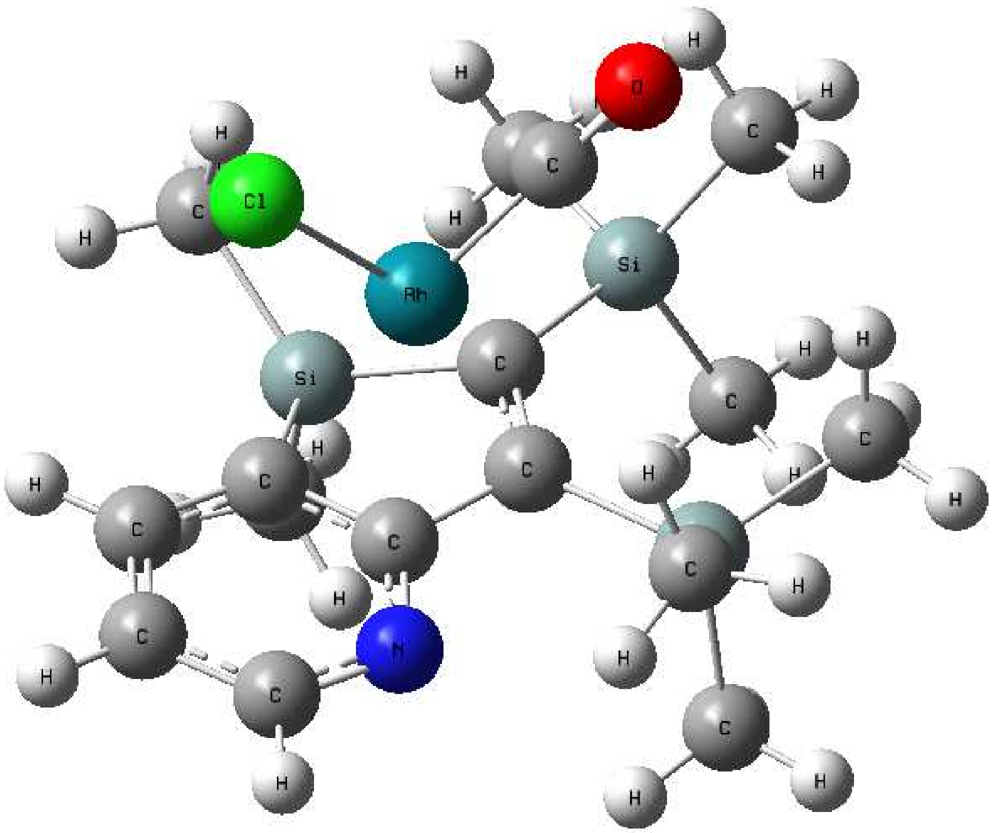

Figure S21. Optimized structure of **model 3(1)** corresponding to combined structure of compound **3** and RhCl(CO).

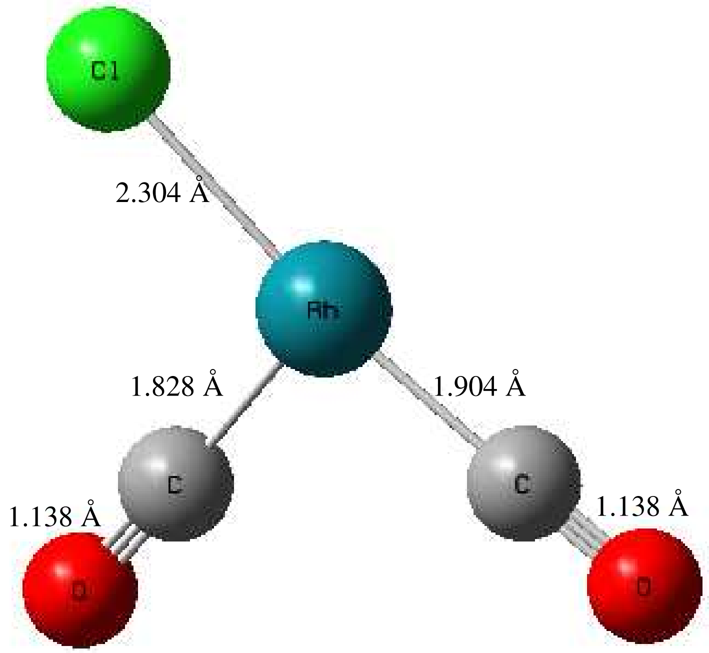

Figure S22. Optimized structure of $RhCl(CO)_2$.

From **Figures S23** to **S29**, LM and TS structures on trans route with $RhCl(CO)_2$ model are drawn. For clarity of figures, H atoms are omitted in the drawing.

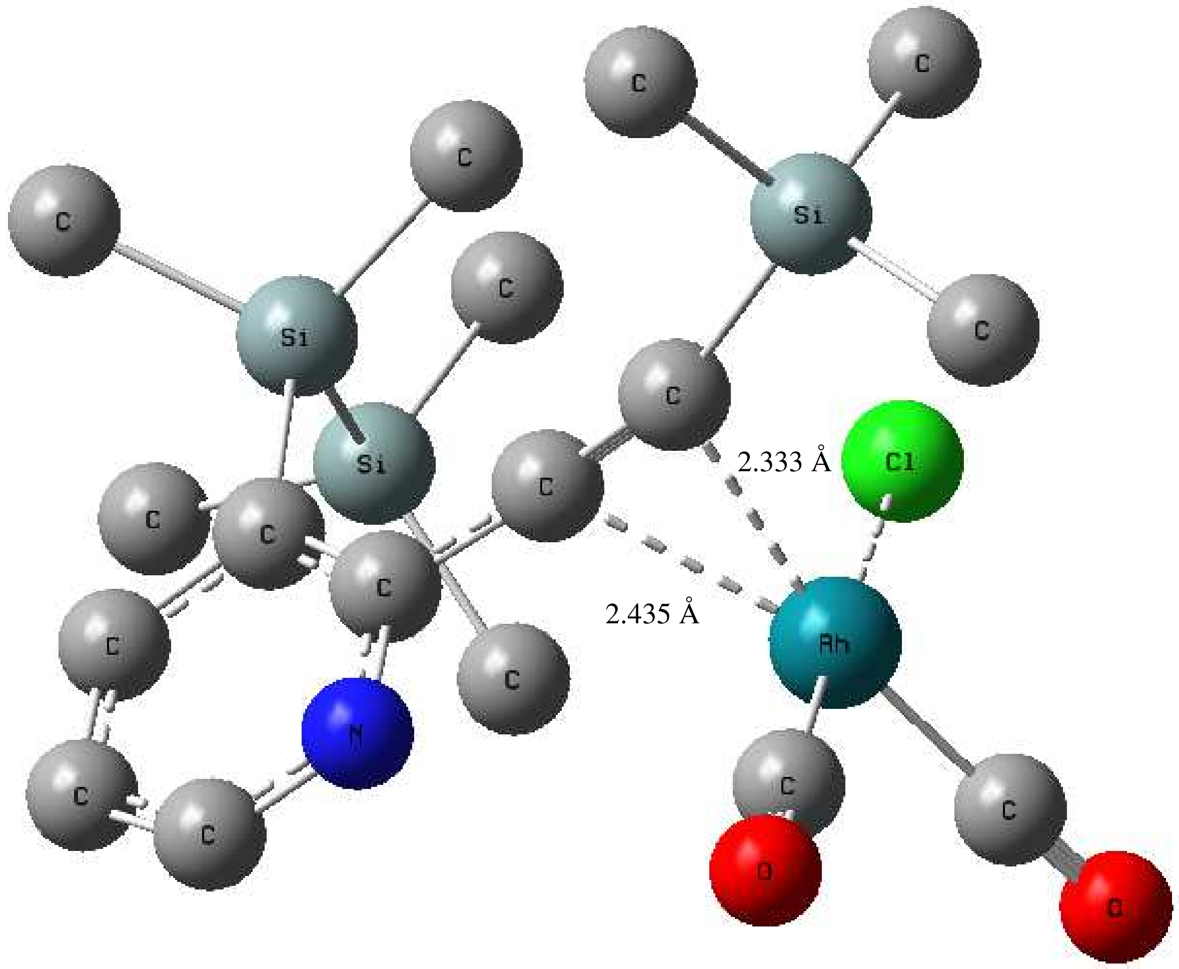

Figure S23. Optimized structure of **model 0(2)**. **Model 0(2)** is a combined structure of **1** and $RhCl(CO)_2$.

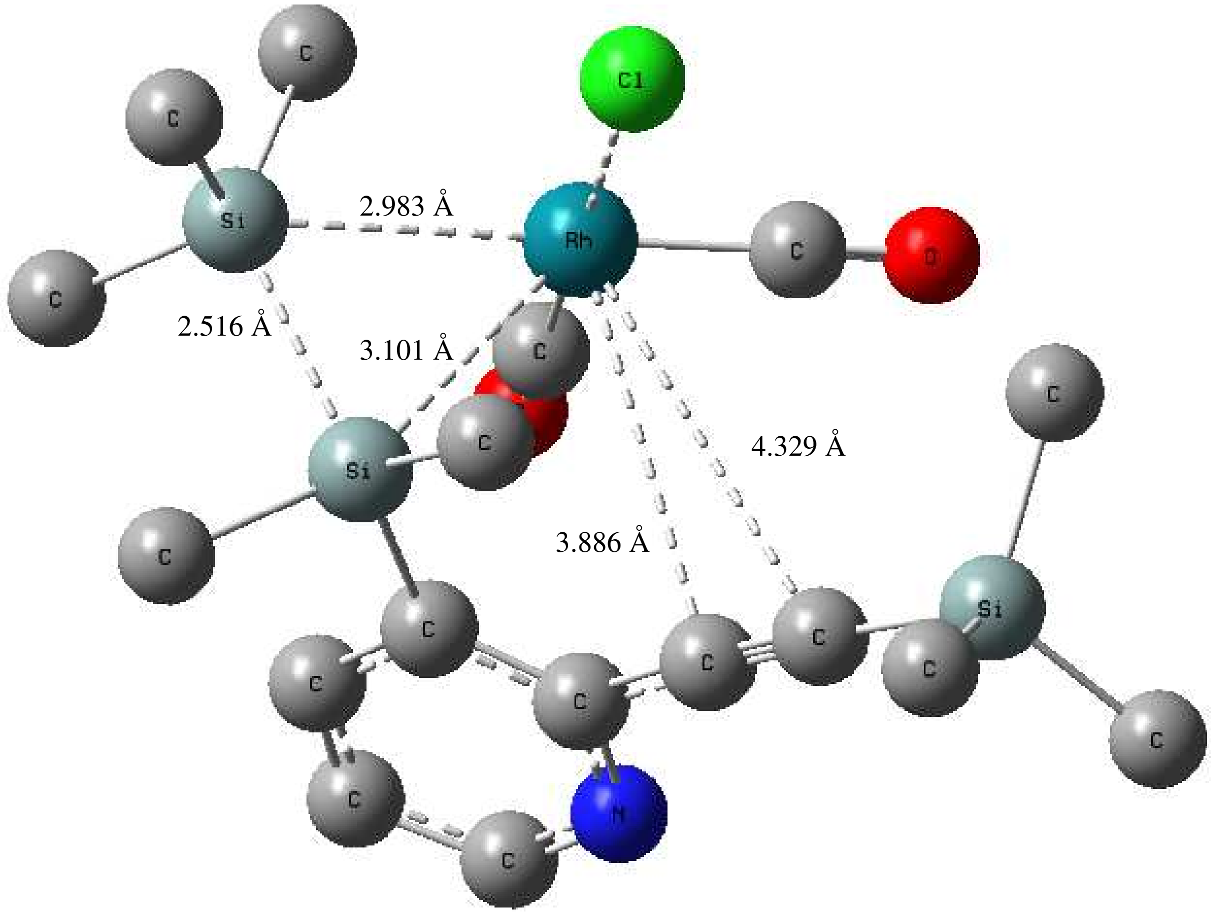

Figure S24. Optimized structure of **TS 0-1(2)**. **TS 0-1(2)** is the TS between **model 0(2)** and **model 1(2)** with $RhCl(CO)_2$ model.

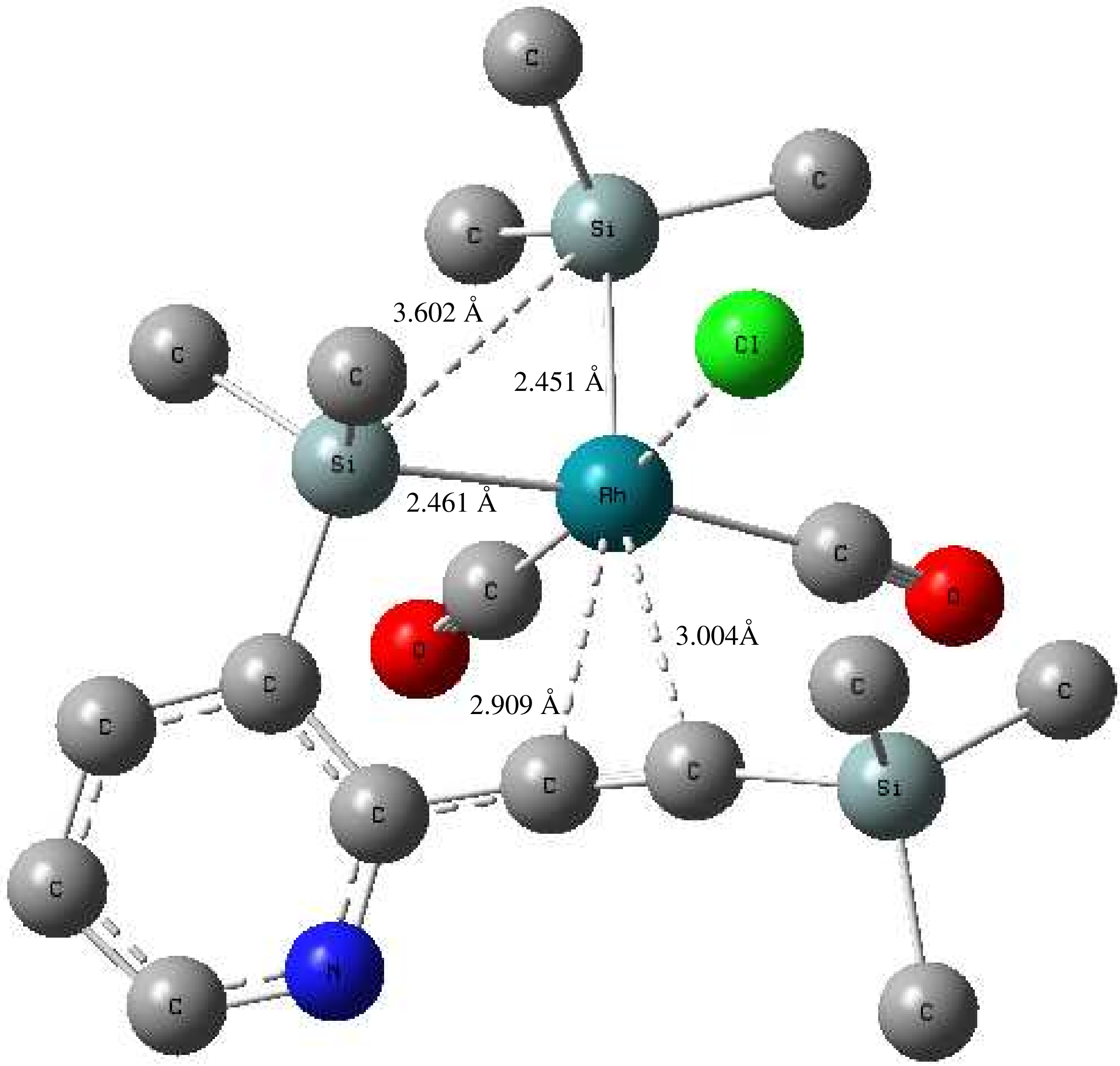

Figure S25. Optimized structure of **model 1(2)** with $RhCl(CO)_2$ model.

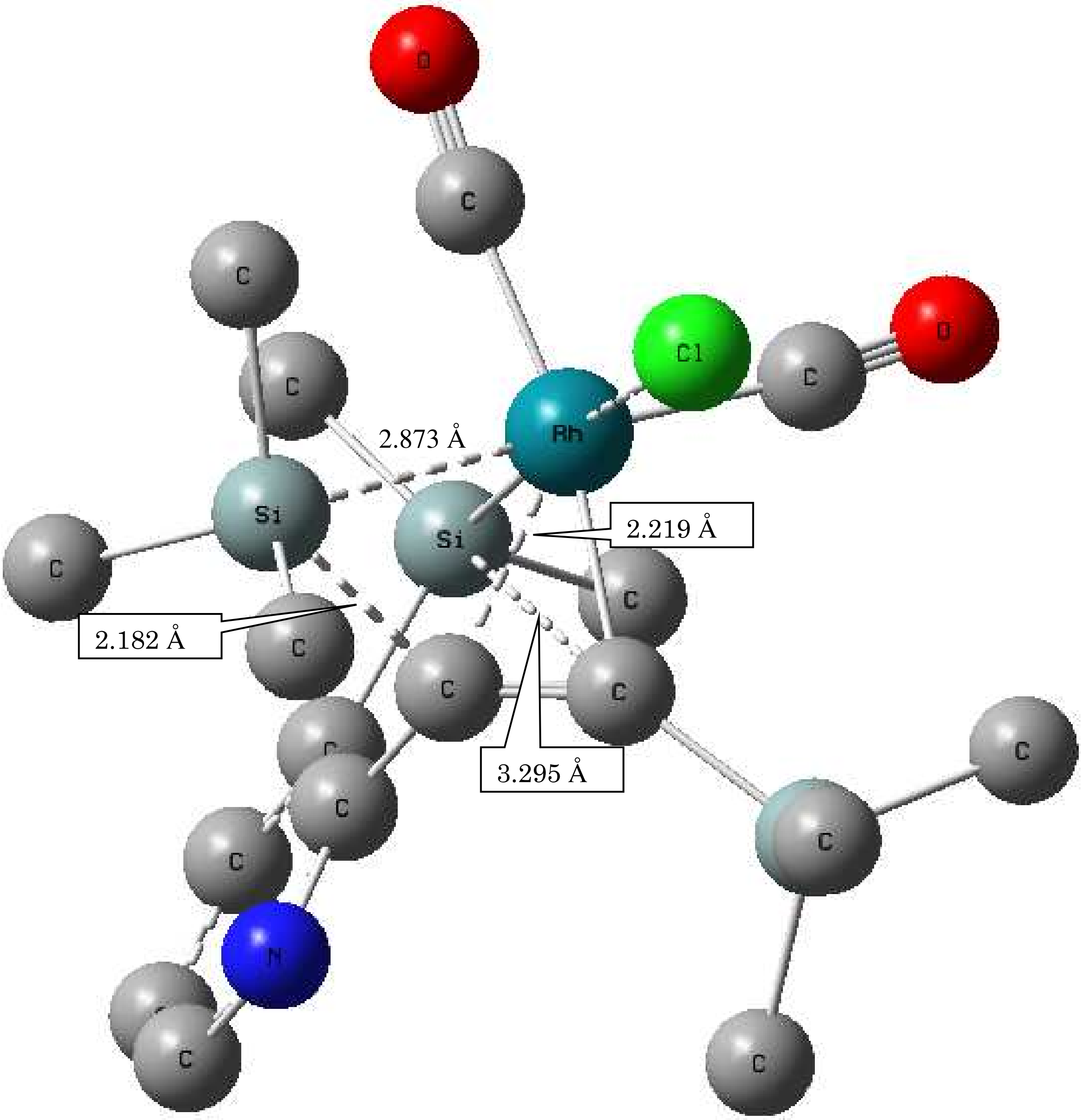

Figure S26. Optimized structure of **TS 1-2(2)**. **TS 1-2(2)** is the TS between **model 1(2)** and **model 2(2)** with $RhCl(CO)_2$ model.

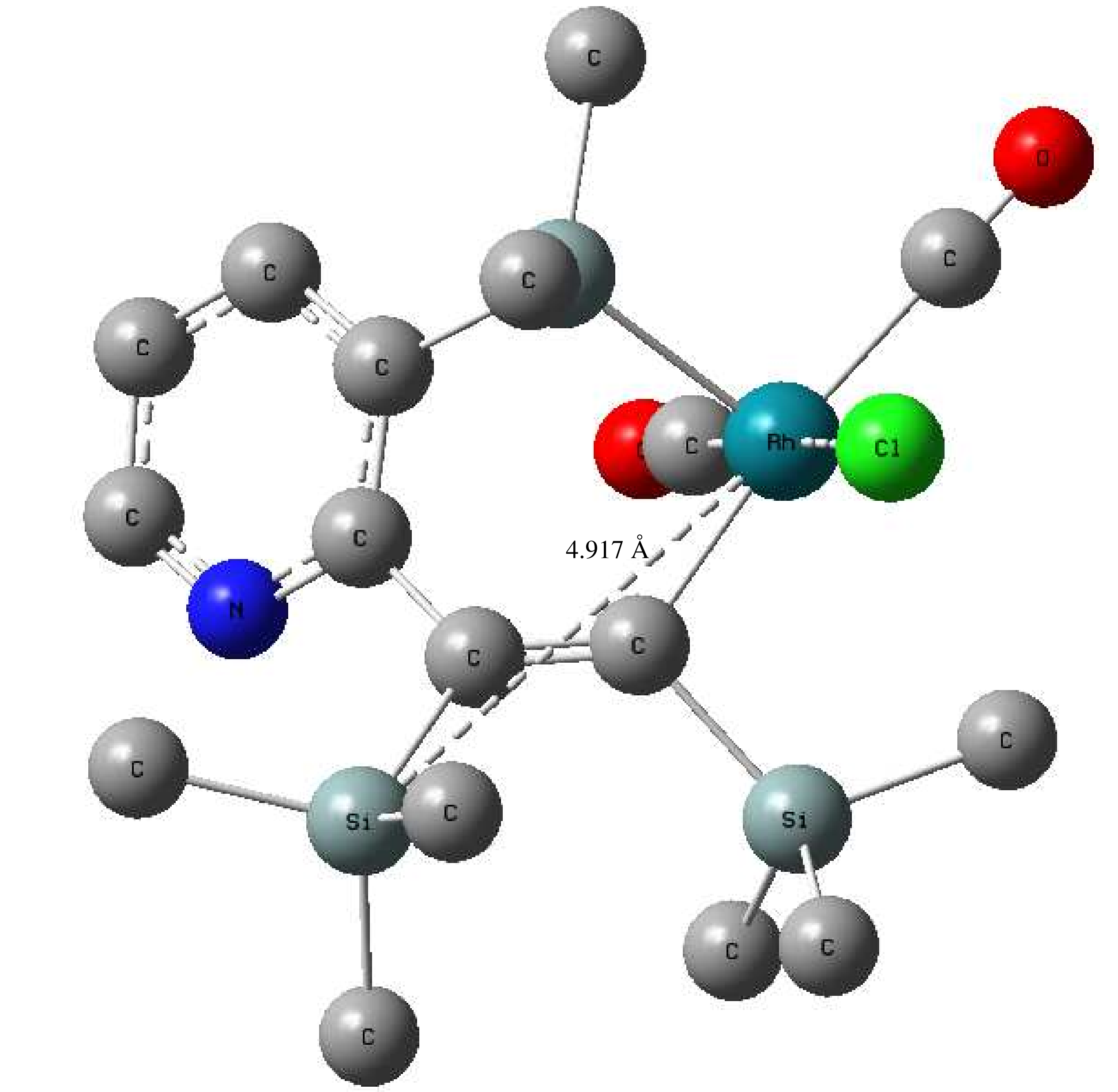

Figure S27. Optimized structure of **model 2(2)** with $RhCl(CO)_2$ model.

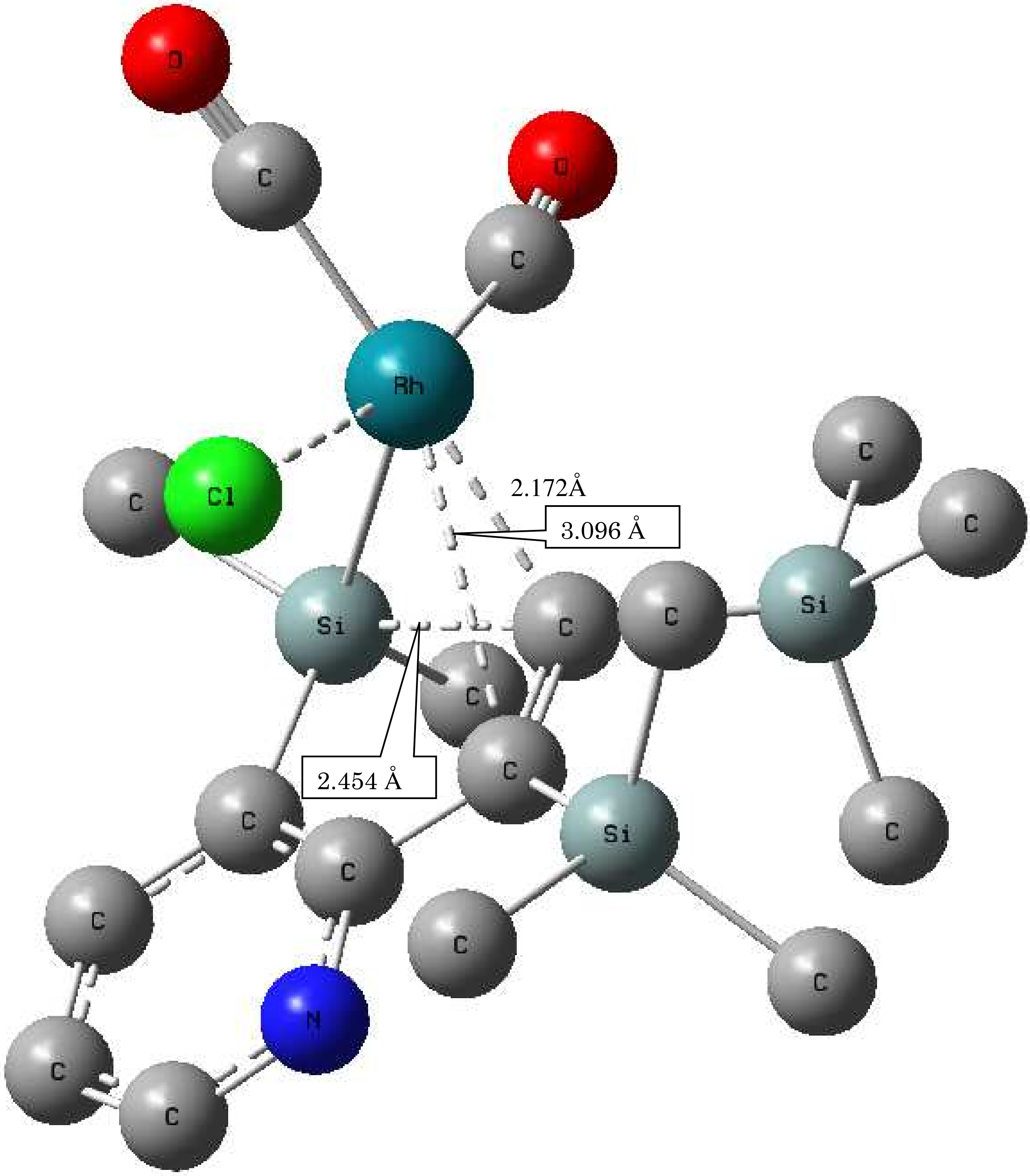

Figure S28. Optimized structure of **TS 2-3(2)**. **TS 2-3(2)** is the TS between **model 2(2)** and **model 3(2)** with $RhCl(CO)_2$ model.

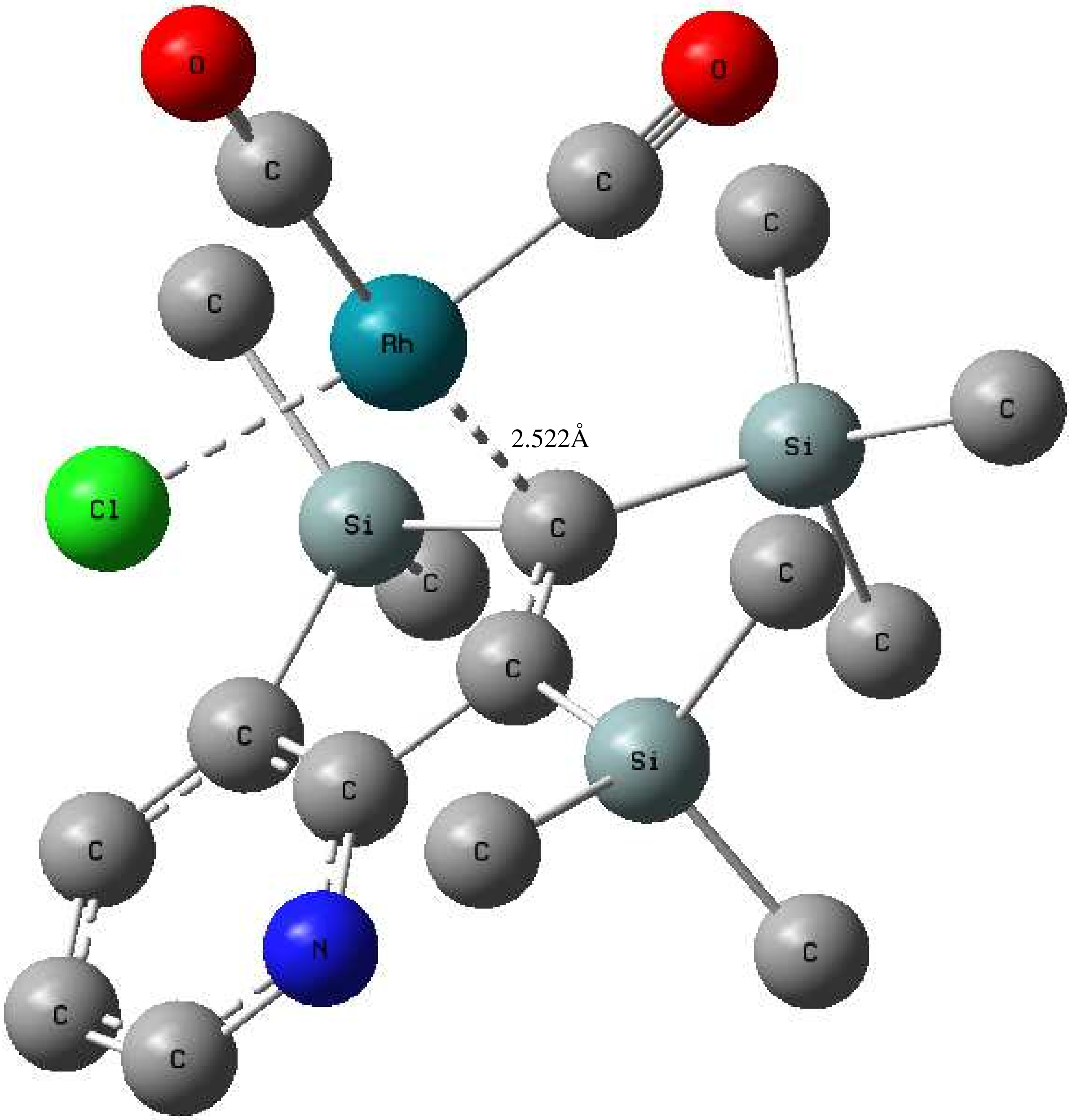

Figure S29. Optimized structure of **model 3(2)** with $RhCl(CO)_2$ model.

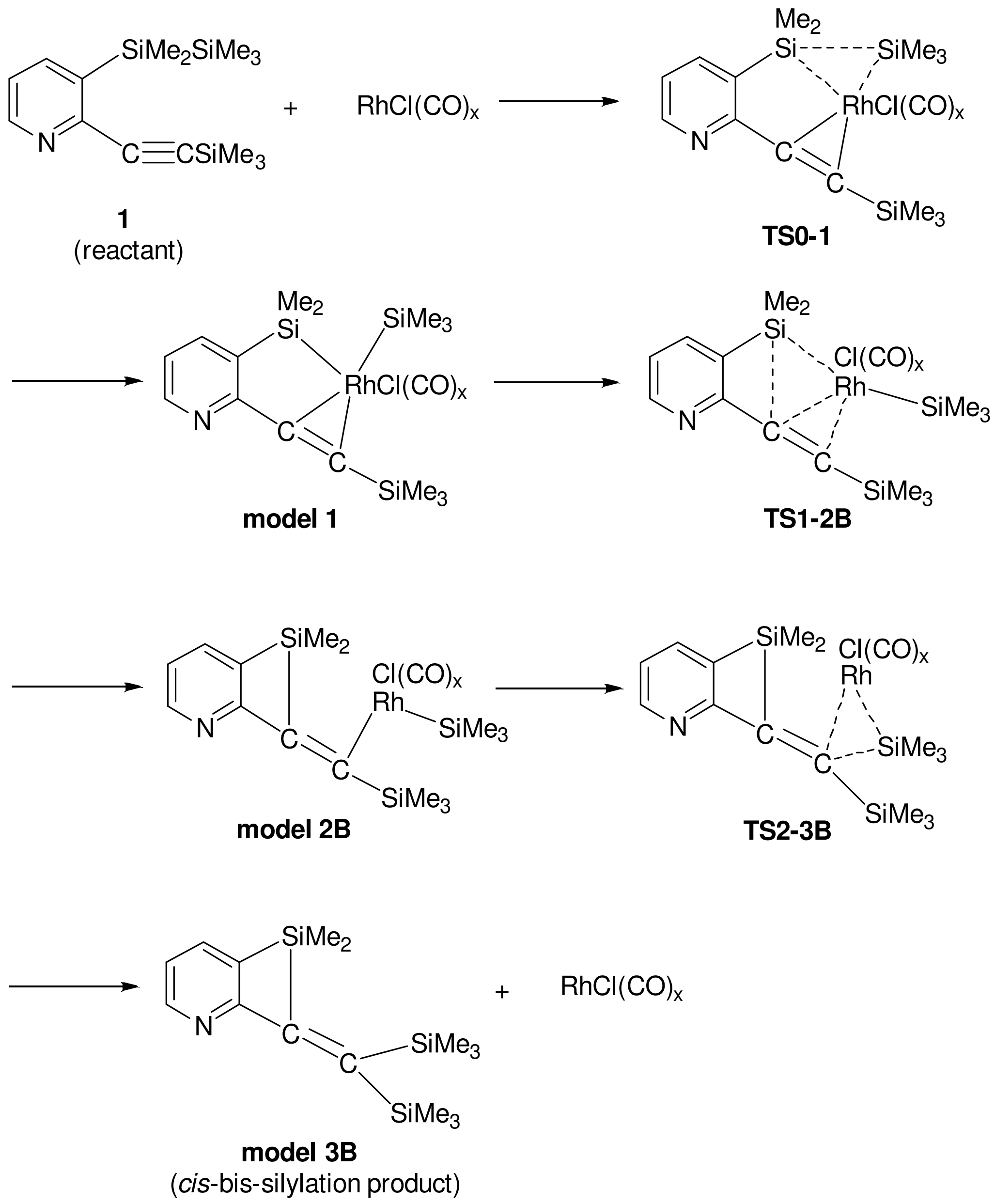

Scheme S1. Proposed Mechanism for Production of *cis*-bis-silylation product. The structures from **model 0** to **model 1** are common to *trans*-bis-silylation product formation route. Each LM and TS names are discriminated by adding suffix "B" from those on *trans*-bis-silylation product formation route.

From **Figures S30** to **S33**, LM and TS structures on cis route with RhCl(CO) model are drawn.

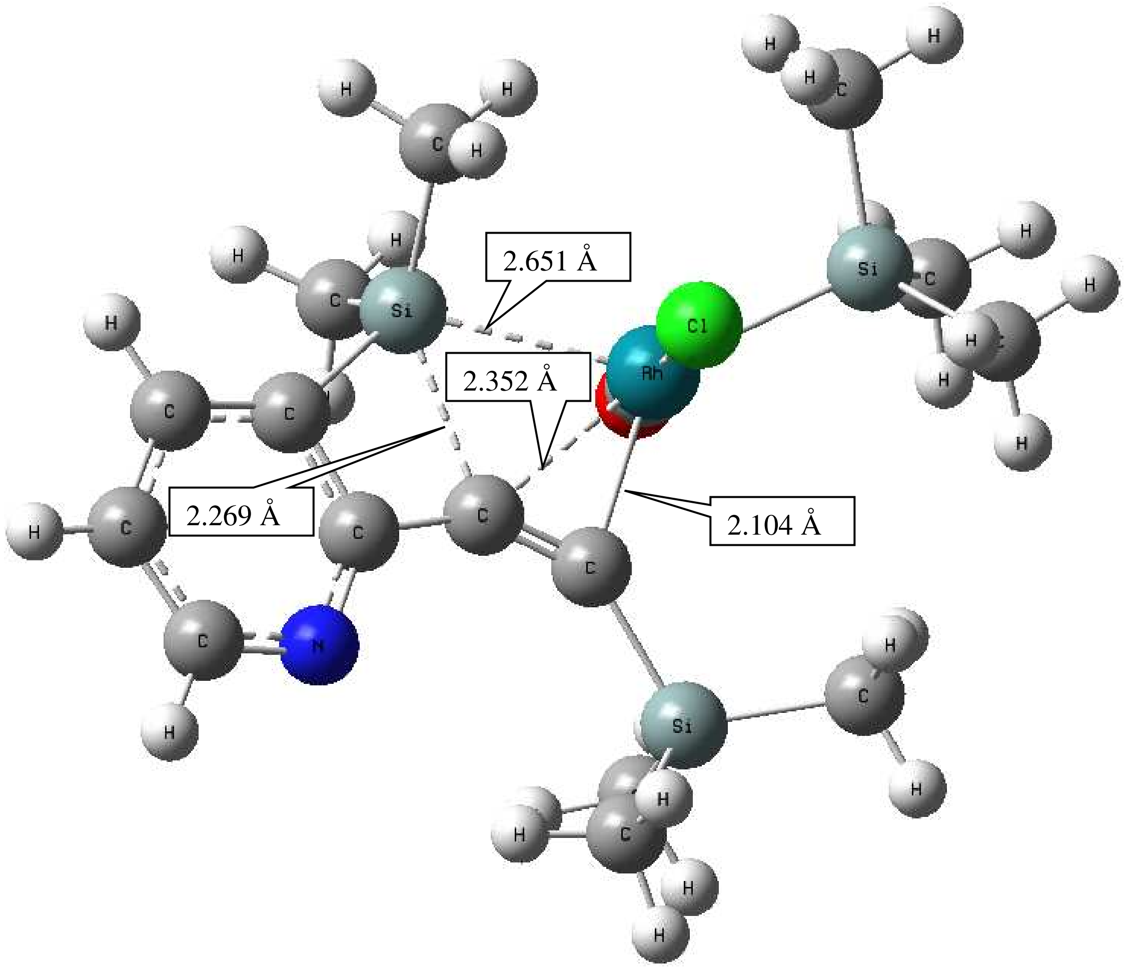

Figure S30. Optimized structure of **TS 1-2B(1)** with RhCl(CO) model. **TS 1-2B(1)** is the TS between **model 1(1)** and **model 2B(1)**.

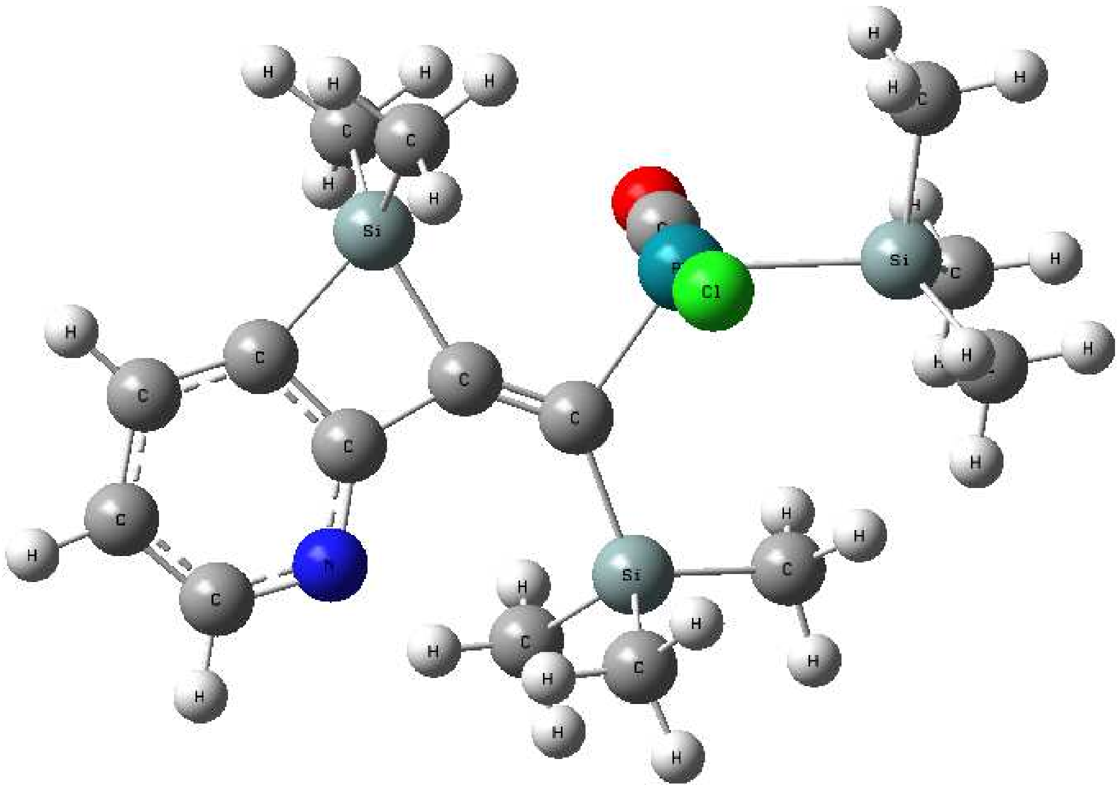

Figure S31. Optimized structure of **model 2B(1)** with RhCl(CO) model.

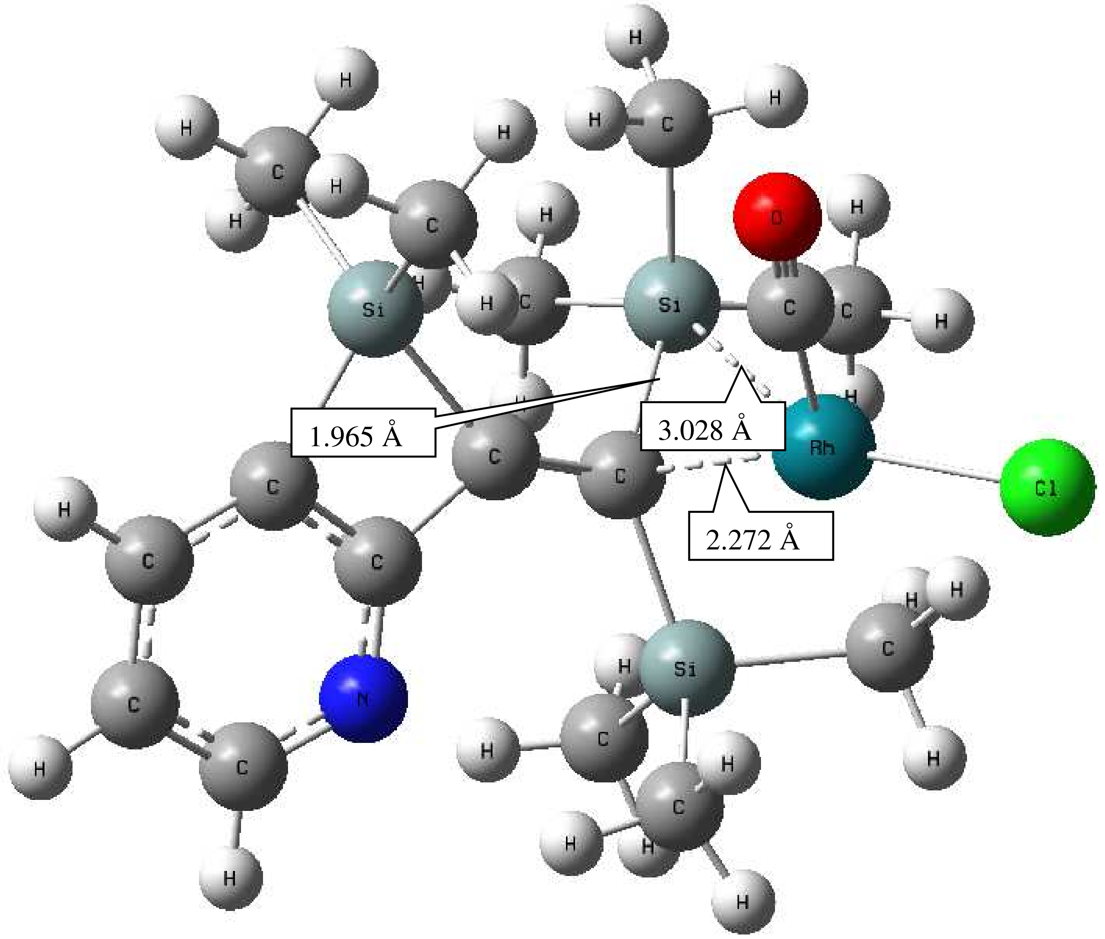

Figure S32. Optimized structure of **TS 2-3B(1)** with RhCl(CO) model. **TS 2-3B(1)** is the TS between **model 2B(1)** and **model 3B(1)**.

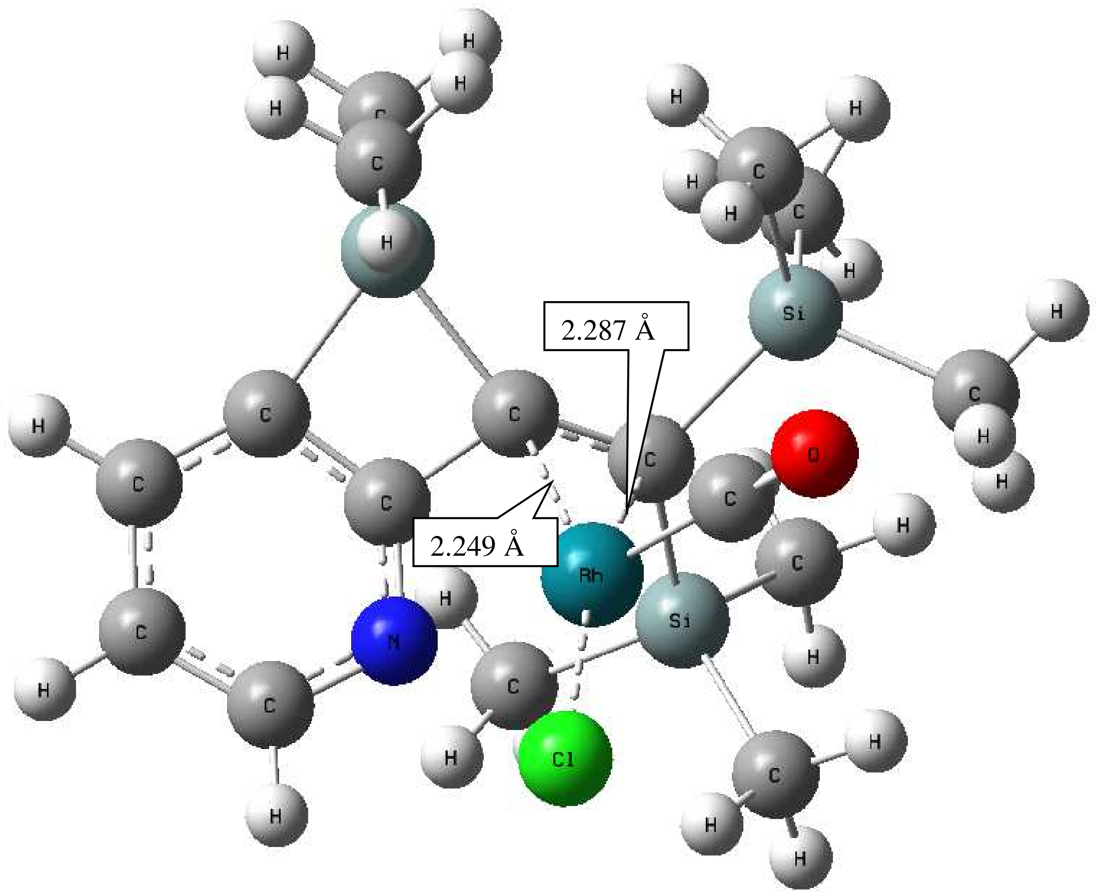

Figure S33. Optimized structure of **model 3B(1)** corresponding to combined structure of *cis*-bis-silylation product and RhCl(CO).

From **Figures S34** to **S37**, LM and TS structures on cis route with $RhCl(CO)_2$ model are drawn. For clarity of figures, H atoms are omitted in the drawing.

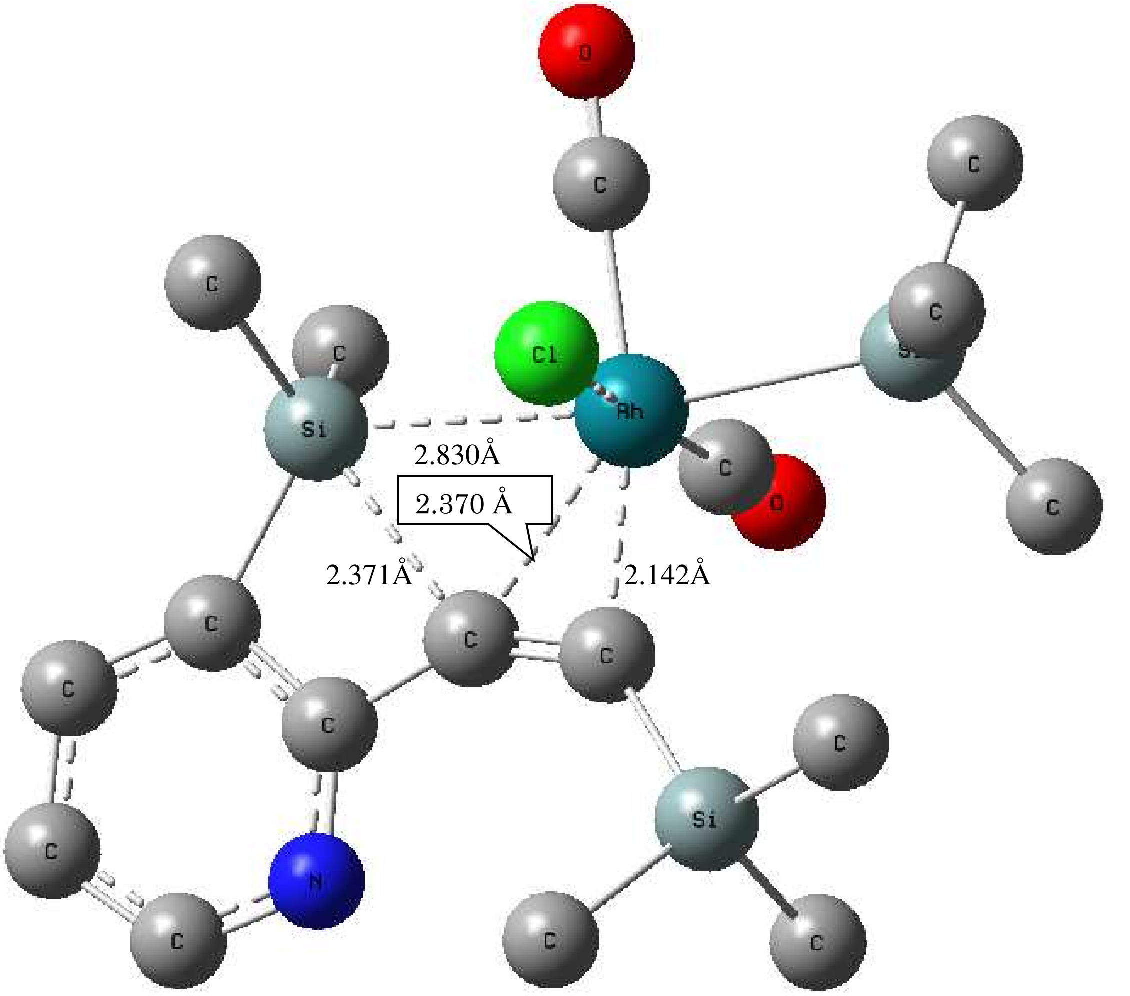

Figure S34. Optimized structure of **TS 1-2B(2)** with $RhCl(CO)_2$ model. **TS 1-2B(2)** is the TS between **model 1(2)** and **model 2B(2)**.

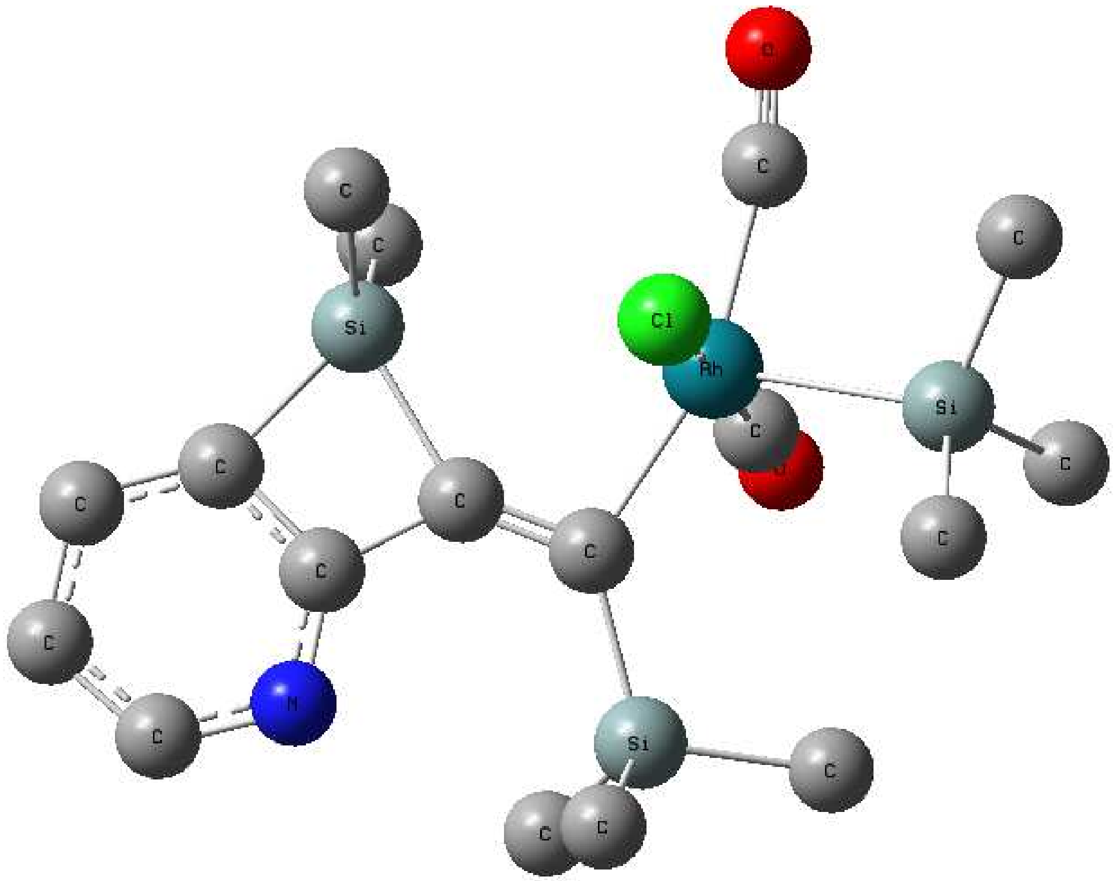

Figure S35. Optimized structure of **model 2B(2)** with $RhCl(CO)_2$ model.

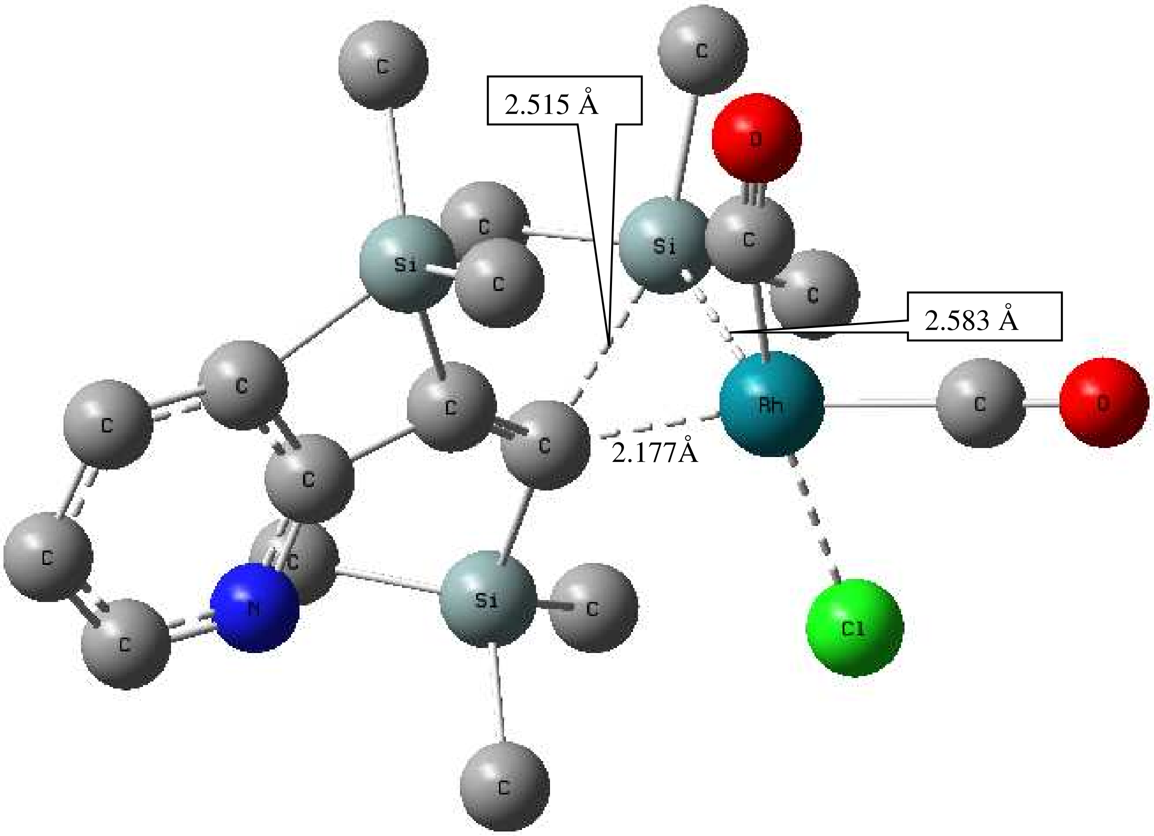

Figure S36. Optimized structure of **TS 2-3B(2)** with $RhCl(CO)_2$ model. **TS 2-3B(2)** is the TS between **model 2B(2)** and **model 3B(2)**.

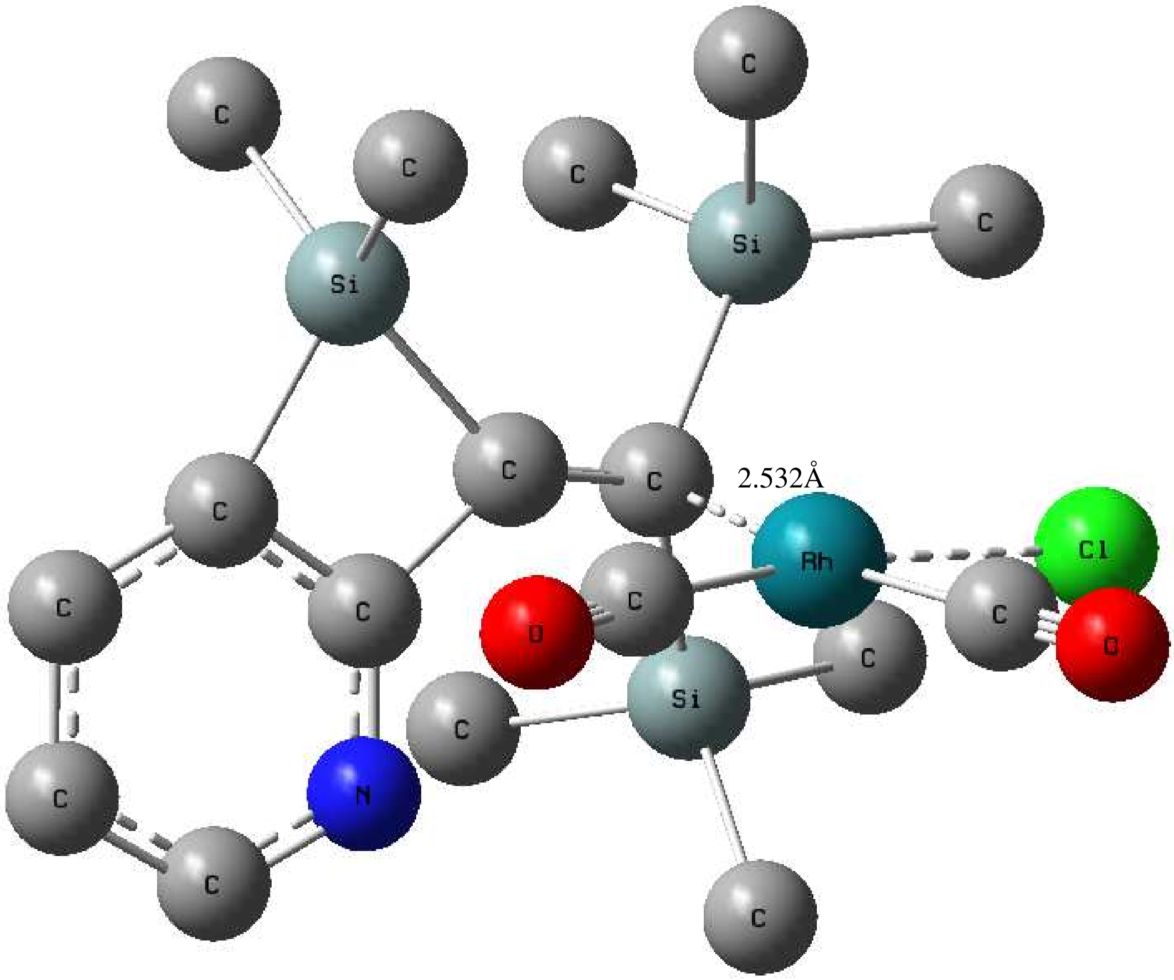

Figure S37. Optimized structure of **model 3B(2)** corresponding to combined structure of *cis*-bis-silylation product and $RhCl(CO)_2$.
